# *Trichinella spiralis* serine protease mediates larval invasion of gut epithelium via binding to CK8 and activating RhoA/ROCK1 pathway

**DOI:** 10.1371/journal.pntd.0013725

**Published:** 2025-11-13

**Authors:** Wen Wen Zheng, Xin Zhuo Zhang, Pei Kun Cong, Ru Zhang, Shao Rong Long, Xi Zhang, Ruo Dan Liu, Zhong Quan Wang, Jing Cui

**Affiliations:** Department of Parasitology, School of Basic Medical Sciences, Zhengzhou University, Zhengzhou, China; Zhejiang Wanli University, CHINA

## Abstract

**Background:**

*Trichinella spiralis* adult worms and larvae parasitize respectively in small intestine and skeletal muscles of the same host, and larval invasion of gut mucosa is the pivotal step for *T. spiralis* infection. A *T. spiralis* serine proteinase (TsSPc) was identified in its intestinal infective larva (IIL) ES antigens. TsSPc is involved in larval invasion of gut epithelium, but the mechanism is not completely elucidated. The purpose of this study was to investigate the mechanism of TsSPc action in larval invasion of gut mucosa.

**Methodology/principal finding:**

The results of molecular docking, immunofluorescence assay (IFA), GST pull-down and co-immunoprecipitation (Co-IP) showed that rTsSPc specifically bound to cytokeratin 8 (CK8) receptor in Caco-2 cells and activated RhoA/ROCK1 signaling pathway, as demonstrated by the evidently increased expression levels of CK8, RhoA and ROCK1. The results of qPCR and Western blot analysis revealed that binding of rTsSPc with CK8 and activation of RhoA/ROCK1 pathway significantly decreased the expression levels of gut epithelial tight junctions (TJs, E-cad, Occludin and Claudin-1), and increased the paracellular permeability. Knocking down CK8 in Caco-2 cells and ROCK1 pathway inhibitor Y27632 obviously inhibited the activation of RhoA/ROCK1 pathway, abolished rTsSPc-decreased TJs expression, rTsSPc-increased paracellular permeability, and inhibited larval invasion of Caco-2 monolayers *in vitro*. When the mice were pretreated with CK8 inhibitor Dasatinib and ROCK1 pathway inhibitor Y27632, and then orally infected with *T. spiralis* larvae, the activation of CK8 and RhoA/ROCK1 was significantly suppressed, intestinal permeability and adult worm burdens were obviously decreased, and intestinal inflammation was also distinctly alleviated. The number of intestinal goblet cells, expression of mucins (Muc2 and Muc5ac) and inflammatory cytokines (IL-1β, TNF-α, IL-10 and TGF-β) were significantly reduced.

**Conclusions:**

TsSPc binding to CK8 receptor in gut epithelium activated RhoA/ROCK1 pathway, reduced TJs expression and disrupted gut epithelial integrity, therefore mediated larval invasion of host gut mucosa. TsSPc might be considered as a promising vaccine molecular target for intercepting *T. spiralis* invasion and infection.

## Author summary

A *Trichinella spiralis* serine proteinase (TsSPc) was identified in its intestinal infective larva (IIL) ES antigens. The purpose of this study was to investigate the mechanism of TsSPc action via binding to CK8 receptor in larval invasion of enteral mucosa. The results of molecular docking, immunofluorescence assay (IFA), GST pull-down and co-immunoprecipitation (Co-IP) confirmed that rTsSPc specifically bound to CK8 receptor in Caco-2 cells and activated RhoA/ROCK1 signaling pathway, as demonstrated by the evidently increased expression levels of CK8, RhoA and ROCK1. rTsSPc binding with CK8 and RhoA/ROCK1 pathway activation significantly decreased the expression levels of gut epithelial tight junctions (TJs, E-cad, Occludin and Claudin-1), and increased the paracellular permeability. Knocking down CK8 in Caco-2 cells and ROCK1 inhibitor Y27632 obviously inhibited the activation of RhoA/ROCK1 pathway, abolished rTsSPc-reduced TJs expression, rTsSPc-increased paracellular permeability, and inhibited larval invasion of Caco-2 monolayers. In the *T. spiralis*-infected mice pretreated with CK8 inhibitor and ROCK1 pathway inhibitor, the activation of CK8 and RhoA/ROCK1 was significantly suppressed, intestinal permeability and adult worm burdens were obviously decreased, and intestinal inflammation was also distinctly alleviated. These findings indicated that TsSPc binding to CK8 in gut epithelium activated RhoA/ROCK1 pathway, reduced the TJs expression and damaged intestinal epithelial integrity and barrier function, therefore mediated larval invasion of host intestinal mucosa. TsSPc might be considered as a promising vaccine molecular target for intercepting *T. spiralis* invasion and infection.

## Introduction

Trichinellosis is one of the serious zoonotic parasitic diseases distributed globally [1]. *Trichinella* infection occurs via consuming raw meat or undercooked meat that contains infectious *Trichinella* larvae [2]. From 2009 to 2020, a total of eight outbreaks of human trichinellosis involving 479 cases and two deaths were reported in China, and seven of 8 outbreaks (87.50%) were caused by the consumption of raw or semi-cooked pork [3]. Only in 2023, 76 confirmed human cases of trichinellosis were reported by 11 member states of the European Union [4]. *Trichinella* infection is not only a significant public health issue but also a major food safety concern for meat products. Therefore, it is necessary to develop a preventive anti-*Trichinella* vaccine to control *Trichinella* infection in food animals.

After *T. spiralis* muscle larvae (ML) encapsulated in skeletal muscles are digested by gastric juice, the ML are released from their collagen capsules and become intestinal infective larvae (IIL) [5,6]. The IIL then invade intestinal epithelium where they undergo four molts and develop into adult worms (AW) [7]. After being mated in intestinal mucosal epithelium, adult worms produce newborn larvae (NBL). These NBL subsequently penetrate the capillaries to enter the bloodstream, migrate to the skeletal muscles, and develop into the encapsulated ML, thus completing the *T. spiralis* life cycle [8]. The IIL invasion of intestinal mucosa is the prior procedure of *T. spiralis* infection, and the binding and interaction between *T. spiralis* molecules and intestinal epithelial cells (IECs) are crucial to its pathogenesis [9]. However, the mechanism of larval invasion has not yet been fully elucidated [10].

*T. spiralis* serine protease (TsSP) is a member of the serine protease family, characterized by a key serine residue in its active center, which endows the enzyme with the ability to catalyze protein hydrolysis reactions [11]. This enzyme can hydrolyze the extracellular matrix (ECM) and intercellular junction proteins in host tissues, facilitating the *T. spiralis* larval penetration of intestinal mucosa and muscle tissues. Its activity plays a crucial role in the larval initial infection and migration in host [12]. Serine protease might be the major virulence factor of *T. spiralis* larvae to invade the host. It reduced the contents of intestinal epithelial tight junctions (TJs) through activating p38-MAPK pathway, thereby affecting intestinal barrier integrity [13]. Yang et al. used RNA interference (RNAi) technology to verify the important role of TsSP in the *T. spiralis* life cycle. RNAi knockdown of TsSP gene significantly reduced the expression level and enzymatic activity of TsSP, inhibited larval invasion of IECs, and decreased female reproductive capacity [14,15].

In previous studies, a *T. spiralis* serine proteinase (TsSPc, GenBank: AAD09211.1) was identified in the IIL excretory/secretory (ES) proteins. TsSPc is a secreted protein with strong antigenicity and is expressed in various worm stages of the parasite. It is primarily localized in the cuticle, stichosome and embryos. Recombinant TsSPc (rTsSPc) specifically bound to IECs. The results of GST pull-down, mass spectrometry (MS) and Co-IP tests showed that rTsSPc specifically bound and interacted with the receptor for activated C kinase 1 (RACK1) in Caco-2 cells. rTsSPc activated the MAPK/ERK1/2 pathway, reduced the expression of TJs (Occludin, Claudin-1 and E-cad), and significantly increased paracellular permeability, thereby mediated the larval invasion of gut mucosa. Nevertheless, while anti-rTsSPc antibodies and TsSPc-specific dsRNA were used in the *in vitro* larval invasion test, only partial larval invasion was restrained; when the RACK1 receptor and ERK1/2 pathway inhibitors (harringtonolide and PD98059) were preadministered in mice, larval invasion of gut mucosa was partially inhibited, but not impeded fully [16,17]. These findings suggested that the mechanism of TsSPc disrupting enteral epithelial integrity and promoting larval invasion is not fully clear. Larval invasion of gut mucosa might be involved in other mechanisms (activation of other receptors and signal pathways).

Additionally, our previous results of mass spectrometry on GST pull-down assays showed that type II cytokeratin 8 (CK8) in Caco-2 cells was identified as a binding partner of rTsSPc. But the CK8 biological function in *T. spiralis* invasion and infection is still unclear [17]. Keratins are a family of structural proteins that form the intermediate filaments of the cytoskeleton in epithelial cells. They are among the most abundant cytoskeletal proteins, accounting for 5% of the total IEC proteins. Thick external barriers, such as the skin, mainly express CK1 and CK5 (Type II) as well as CK10 and CK14 (Type I), while internal epithelia, such as the intestines, primarily express CK8 (Type II) and CK18 (Type I) [18]. Keratins are crucial for tissue integrity and are involved in intracellular signaling pathways, regulating cellular responses to damage, cell growth, and cell death [19]. In the inner epithelium, keratin 8/18 promotes cell rigidity by activating the ROCK signaling pathway through cortical actin [20]. The increased CK8 expression reduced the strength of Claudin-1 and Occludin and caused structural disruption. The expression levels of F-actin, Occludin, and Claudin-1 were downregulated, while the Ras homolog (RhoA) activity was increased. Conversely, silencing of CK8 blocked the effects of decreased F-actin expression levels and increased RhoA activity [21].

The aim of this study was to investigate the role and molecular mechanism of TsSPc binding to gut epithelium CK8 receptor in *T. spiralis* larval invasion of host gut mucosa. The prospective results will be helpful to further understand the mechanism of *T. spiralis* invasion and pathogenesis; they also provide the basis to develop a novel anti-*Trichinella* vaccine against intestinal invasive stage worms.

## Materials and methods

### Ethics statement

This research was carried out in strict compliance with the National Guidelines for Experimental Animal Welfare, which was issued by the Ministry of Science and Technology of the People’s Republic of China in 2006. Moreover, all animal-related experimental procedures in this study were formally approved by the Life Science Ethics Committee of Zhengzhou University, with the approval number ZZUIRB GZR 2021–0044.

### *Trichinella* species, experimental animals and cells

*Trichinella spiralis* was the laboratory-passaged isolate (ISS534) from a naturally infected pig in Henan province of China. Experimental animals (4–6 weeks old female BALB/c mice) used in this study were purchased from the Henan Provincial Experimental Animal Center (Zhengzhou, China). Human colorectal adenocarcinoma epithelial cell line (Caco-2) was maintained and preserved in our laboratory.

### Cell culture

To obtain sufficient cells to prepare cell monolayer, Caco-2 cells were initially seeded in T25 flasks (NEST, Wuxi, China) containing Dulbecco’s Modified Eagle Medium (DMEM) (Servicebio, Wuhan, China) and cultured for 7 d. The medium was supplemented with 10% fetal bovine serum (FBS) (Gibco), 100 U/ml penicillin, 100 µg/ml streptomycin, and 100 mM nonessential amino acids (Solarbio, Beijing, China). After being cultured for 7 d, the cells were digested with trypsin and then cultured again at 37 ℃ and 5% CO₂ for 14–21 d until a cell monolayer was formed on a cover slide [9].

### Collection of intestinal *T. spiralis* worms and excretory-secretory (ES) antigens

The IIL ES antigens were prepared as described previously. Briefly, the IIL were collected from the intestines of mice infected with *T. spiralis* ML (5000 ML per mouse) at 6 hours post infection (hpi) [22]. IIL worms were thoroughly washed with sterile normal saline and RPMI 1640 medium without serum containing 100 U/ml penicillin and 100 µg/ml streptomycin. And then, the worms were cultured at a density of 5000 worms/ml in a 37 ℃, 5% CO₂ incubator for 18 h. The culture supernatant was collected and concentrated using an Amicon Ultra3 centrifugal filtration device (MW cut-off value: 3 kDa). The concentrated supernatant was then centrifuged at 4 ℃ and 5000 × *g* for 2 h. The IIL ES antigens were collected, and protein concentration was assessed and stored at -80 ℃ [23].

### Preparation of rTsSPc with a GST-tag

rTsSPc with a His-tag was prepared in our laboratory and exhibited a good antigenicity [16]. To carry out the subsequent GST pull-down assay, rTsSPc with a GST-tag was also expressed and purified in this study. Briefly, full-length cDNA sequence of TsSPc gene (GenBank: U62659.1) was cloned into the pGEX-4T-1 vector, and the recombinant expression plasmid pGEX-4T-1/TsSPc was constructed. The recombinant pGEX-4T-1/TsSPc was subsequently introduced into *E. coli* Origami (Novagen), and the expression of rTsSPc was induced with 0.5 mM isopropyl β-D-1-thiogalactopyranoside (IPTG) at 16 °C for duration of 3 d. The rTsSPc with a molecular weight (MW) of 51.18 kDa was subsequently purified using GST-Sepharose Resin 4FF (Settled Resin) (Sangon Biotech, Shanghai, China) [24,25]. SDS-PAGE results showed that the MW of purified rTsSPc was 51.18 kDa, and it was identified on Western blotting with anti-rTsSPc immune serum.

### Molecular docking of TsSPc and CK8

Molecular docking of TsSPc and CK8 was predicted computationally as previously reported [26].The amino acid sequences of TsSPc (GenBank: AAD09211.1) and CK8 (GenBank: NP_001243211.1) were downloaded from the NCBI website (https://www.ncbi.nlm.nih.gov/). The 3D structure of CK8 was predicted by AlphaFold and exported as a PDB file, while the TsSPc structure was obtained from the AlphaFold Protein Structure Database (https://alphafold.ebi.ac.uk/). Both PDB files were submitted to the GRAMM docking server, where rigid-body docking was performed based on an integrated scoring system of geometric, electrostatic, and hydrophobic complementarity. Docking poses were ranked according to their scores, and the best TsSPc–CK8 complex was chosen using the following criteria: highest composite score and PDBePISA validation (buried interface area ≥ 850 Å², dissociation free energy ≤ –10 kcal/mol, and the presence of stable hydrogen bonds and/or salt bridges). PDBePISA was also used to analyze interface parameters, including hydrogen bonds, salt bridges, and binding free energy. The resulting 3D structure was visualized using PyMOL.

### Immunofluorescent assay (IFA) of co-localization of rTsSPc and CK8 in Caco-2 cells

Co-localization of rTsSPc and CK8 in Caco-2 cells was examined by IFA as previously described [7,27]. When Caco-2 cell monolayer was grown to 90% confluence, it was incubated with rTsSPc (20 μg/ml) at 37 °C, 5% CO₂ for 2 h. The IIL ES antigens (20 μg/ml) were served as the positive control, and PBS was used as the negative control [9]. The cells were then fixed with 4% paraformaldehyde for 20 min and permeabilized with 0.25% Triton-X-100 for 10 min. After blocking with 1% bovine serum albumin (BSA) at 37 °C for 1 h, the cells were incubated overnight at 4 °C with primary antibodies: mouse anti-rTsSPc serum (1:10) prepared in our department [16], and anti-CK8 antibody (1:100, Bioss, Beijing, China). Normal murine serum (1:10) was also used as a control for anti-rTsSPc serum to exclude the non-specific binding of mouse antibodies. After washes with PBS, they were incubated with secondary antibodies: Alexa Fluor 488-conjugated goat anti-mouse IgG (1:100, Servicebio) and CY3-conjugated goat anti-rabbit IgG (1:100, Servicebio) at 37 °C for 1 h. The nuclei were counterstained with DAPI (Solarbio, Beijing, China), and the cells were observed and imaged under a fluorescence microscope (Olympus BX51, Tokyo, Japan) [17].

### GST pull‑down and Co‑immunoprecipitation (Co‑IP) assay

To investigate the binding and interaction between rTsSPc and CK8 receptor in Caco-2 cells, the Glutathione S-transferase (GST) pull-down assay was performed as previously described [28,29]. The rTsSPc with GST tag was first incubated with GST resin (Sangon Biotech, Shanghai, China) for 2 h at 4 ℃ to allow their binding. After washes with binding buffer (4.2 mM Na₂HPO₄, 2 mM KH₂PO₄, 140 mM NaCl, and 10 mM KCl), the lysate of normal Caco-2 cells was incubated with the rTsSPc-pre-immobilized on the GST resin for 2 h at 4℃. The binding and interaction were then identified by Western blot with anti-rTsSPc serum (1:100), anti-GST tag antibody (1:1000, Servicebio, China), and anti-CK8 antibody (1:2000, Bioss, China). The GST tag protein and blank beads were set as negative controls [8,30].

To further verify the binding and interaction between rTsSPc and CK8, the Co‑IP assay was also carried out as reported before [31]. After Caco-2 monolayer was co-incubated with rTsSPc with His tag (20 µg/ml) at 37 ℃ and 5% CO₂ for 2 h, the monolayer was lysed by a mild DISC lysis buffer (30 mM Tris-HCl, pH 8.0, 120 mM NaCl, 10% glycerol, 1% Triton X-100) to obtain soluble cellular proteins [17]. Subsequently, Protein A/G agarose beads were incubated with mouse anti-rTsSPc serum and normal IgG by rotation at 4 ℃ for 2 h, followed by the addition of the Caco-2 cell lysates for further incubation for 4 h. After washes, the protein was boiled to be denatured, the beads were used for Western blot analysis, normal mouse IgG coupled with Protein A/G was incubated with Caco-2 cell proteins was served as the negative control [32].

### Real-time quantitative PCR (qPCR) assay

To evaluate the transcription levels of CK8, RhoA, ROCK1 and TJs (E-cad, Occludin and Claudin-1) in Caco-2 cells treated with rTsSPc, CK8 receptor knockdown, and ROCK1 pathway inhibitors (Y27632), the qPCR assay was conducted as previously reported [33,34]. To confirm whether the binding of rTsSPc to CK8 disrupts the TJ integrity by activating the CK8/RhoA/ROCK1 signaling pathway and down-regulating the expression of TJs proteins, si-RNA was used to knock down CK8 (CK8-si-RNA: 5′-GCCUCCUUCAUAGACAAGGUA (dTdT)-3′), and Rho-associated coiled coil-forming protein kinase 1 (ROCK1) signaling pathway inhibitor Y27632 (10 µM; MCE, USA) was also used in this study. Caco-2 cells were treated with 10 µM Y27632 for 24 h, or transfected with 75 µM CK8-si-RNA for 48 h, and then co-incubated with 20 µg/ml of rTsSPc for 2 h, and then total RNAs were extracted from the Caco-2 cells using TRIzol reagent (Sangon, Shanghai, China). The RNA was reversely transcribed into cDNA suitable for qPCR using the Hifair V one-step RT-cDNA digestion SuperMix for qPCR kit (YESEN, Shanghai, China). The cDNA was then amplified using the SYBR Green PCR Master Mix (Servicebio) on the ABI Prism 7500 Fast Sequence Detection System (Applied Biosystems, USA) [35,36]. Specific primers (S1 Table) for qPCR were used to determine the transcription levels of CK8, RhoA, ROCK1, E-cad, Occludin and Claudin-1 in Caco-2 cells and infected murine intestines, and the transcription levels of pro-inflammatory cytokines (TNF-α and IL-1β) and anti-inflammatory cytokines (TGF-β and IL-10) in the infected murine intestines were assessed [37]. Relative transcription levels of the related genes were normalized by subtracting the transcription level of the housekeeping gene Glyceraldehyde-3-phosphate dehydrogenase (GAPDH). The transcription levels of the target genes were calculated using the 2^−ΔΔCt^ method as previously reported [38].

### Western blotting analysis

Caco-2 cells were treated with 10 µM Y27632 (ROCK1 pathway inhibitor) for 24 h and transfected with 75 µM CK8-si-RNA for 48 h, followed by co-incubation with 20 µg/ml rTsSPc for 2 h [39,40]. After Caco-2 cells were lysed with RIPA buffer for 30 min, the lysate was sonicated for 10 seconds and then centrifuged at 12,000 *g* for 20 min to collect the supernatant containing soluble cellular proteins, and the protein concentration of the supernatant was quantified by BCA assay. Subsequently, the proteins were separated by SDS-PAGE and transferred onto a polyvinylidene difluoride (PVDF) membrane in a wet transfer system [41,42]. The membrane was blocked with 5% skim milk at room temperature for 1 h and then incubated overnight at 4 °C with primary antibodies against CK8 (1:2000, Bioss, China), RhoA (1:1000, Zenbio, China), ROCK1 (1:1000, Zenbio, China), E-cad (1:20000), Occludin (1:250), Claudin-1 (1:250) (ThermoFisher, USA), and GAPDH (1:10000, Zenbio, China) with gentle shaking. After three washes with TBST, the membrane was incubated with HRP-conjugated goat anti-rabbit IgG (1:10000) at room temperature for 1 h, followed by three washes with TBST. The reactive bands were visualized using Omni-ECL reagents (Epizyme, Shanghai, China), and the band intensities were analyzed using Image J software (National Institutes of Health, USA) to calculate the relative protein expression levels of related genes [43,44].

### Knockdown of CK8 in Caco-2 cells

Two specific CK8-siRNA sequences were selected: si-CK8–1: 5′-GCCUCCUUCAUAGACAAGGUA (dTdT)-3′; si-CK8–2: 5′- CAGCAUCAUUGCUGAG GUCAA (dTdT)-3′. One nonsense sequence (non-targeting control, si-NC) was also used: 5′-UUCUCCGAACGUGUCACGU (dTdT)-3′). The sequences used in the study have been validated to be the CK8-specific [40,45], and we aligned the siRNA sequences by using the BLAST program to exclude homology with the other genes and to avoid the possible off-target effects. The sequences were synthesized by Sangon Biotech (Shanghai, China). Caco-2 Cells were cultured in a 24-well cell culture plate. When the cell confluence reached 60%, transfection was performed by soaking method with a specific transfection reagent Lipofectamine (e.g., RNATransMate, Sangon, China). Two sterile RNase-free EP tubes were prepared, and DMEM serum-free medium was added to each tube. Then, the siRNA stock solution and RNATransMate were added to the tubes separately, labeling them as tube I and tube II, and gently mixed. After reaction at room temperature for 15 min, the siRNA/RNATransMate transfection complex was produced, and then added to culture plate wells and incubated at 37 °C for 6 h. The transfection status of the cells was observed. If the transfection was successful, the medium was replaced with complete medium and continued to be cultured. Cellular RNA and proteins were extracted for subsequent qPCR and Western blot analysis to detect CK8 expression levels.

### Immunofluorescence assay (IFA) of TJs expression

Previous studies have shown that rTsSPc reduced the expression of TJs proteins [17]. In this study, the inhibitory effects of CK8 knockdown and the pathway inhibitor Y27632 on the rTsSPc-reduced TJs expression were investigated by IFA to verify whether the binding of rTsSPc to CK8 down-regulates TJs proteins by activating the RhoA/ROCK1 signaling pathway. Caco-2 cells were cultured on coverslips in 24-well plates until full confluence. The cells were pre-treated with 10 µM Y27632 for 24 h and transfected with 75 µM CK8-si-RNA for 48 h. Subsequently, 20 µg/ml rTsSPc was added to the cell monolayer and incubated for 2 h. The cells were then washed three times with PBS, fixed with 4% formaldehyde solution for 20 min, and permeabilized with 0.25% Triton X-100 at room temperature for 10 min. The cells were blocked with 10% goat serum at room temperature for 1 h and then incubated overnight at 4 °C with primary antibodies against human E-cad (1:500; Abcam, UK), Occludin (1:160; Invitrogen, USA), and Claudin-1 (1:10; Santa Cruz, USA). Secondary antibodies labeled with CY3 (1:100; Servicebio) were used for 1 h incubation. The cell nuclei were stained with DAPI, and the cells were observed and imaged under a fluorescence microscope (Olympus, Japan) [27,46]. The mean fluorescence intensity of E-cad, Claudin-1 and Occludin in the rTsSPc groups was analyzed by using Image J software.

### Assay of trans-epithelial electrical resistance (TEER) and paracellular permeability

TEER was measured to assess the Caco-2 monolayer barrier damage caused by rTsSPc and the recovery effects of CK8 knockdown and ROCK1 inhibitor. The fluorescent marker used in this study was 4 kDa FITC-dextran (FD4: FITC dextran 4 kDa; Sigma, USA) [17]. Caco-2 cells were cultured in 24-well Trans-well inserts (pore size 0.4 μm) at 37 ℃ and 5% CO₂ for 21 d until full confluence. TEER was measured by Millicell-ERS volt ohmmeter (Millipore, USA) after Caco-2 cells were treated with 10 µM ROCK1 inhibitor Y27632 for 24 h and transfected with 75 µM CK8-si-RNA for 48 h. TEER (% of PBS group) = (test group value - blank group value)/(PBS group value - blank group value) × 100%. Cells were incubated with rTsSPc at 37 °C and 5% CO₂ for 2 h, and TEER was measured.

Paracellular permeability was ascertained by using FD-4. A total of 0.5 mg/ml FD4 solution was added to the upper chamber of the Trans-well system, while PBS was added to the lower chamber. After incubation for 2 h, the solution in the lower chamber was collected and added to a black 96-well plate, and the fluorescence intensity was assessed at an excitation wavelength of 492 nm and an emission wavelength of 520 nm [46]. The fluorescence intensity values of the samples were converted into FD-4 concentrations via a standard curve and compared with the PBS control group [13,47]. Each experiment of both TEER and permeability assays had three biological replicates.

### The *in vitro* larval invasion test

An *in vitro* larval invasion test was performed as reported before [48,49]. Caco-2 cells were seeded in 12-well plates and cultured until 80% confluence. The cells were first treated with 10 µM ROCK1 inhibitor Y27632 for 24 h, or transfected with 75 µM siRNA-CK8 for 48 h. One hundred muscle larvae were activated into the IIL with 5% swine bile for 2 h at 37 ℃ [50,51]. The IILs were washed 20 times with sterile PBS to completely remove the bile. The Caco-2 cell monolayer was overlaid with 100 IIL suspended in semisolid medium, and incubated in 5% CO_2_ for 2 h at 37 ℃ [52]. The number of invaded larvae was observed and numbered under a microscope [41]. The larvae that penetrated into the cell monolayer and actively migrated within the monolayer were defined as invaded larvae; whereas the larvae that were still suspended in culture medium and exhibited the spirally coiled on the surface of the monolayer were defined as non-invaded larvae [25]. The suppressive effect of CK8 knockdown and RhoA/ROCK1 pathway inhibitors on larval invasion of Caco-2 monolayer was evaluated according to the invaded larval number in various groups.

### Animal challenge experiment

In this study, the CK8 inhibitor Dasatinib (DAS) (20 mg/kg) and the ROCK1 pathway inhibitor Y27632 (5 mg/kg) were used to further verify whether the rTsSPc-increased intestinal epithelial permeability is achieved by binding to the CK8 receptor and activating the RhoA/ROCK pathway in intestinal epithelial cells [53]. A total of 75 female BALB/c mice were randomly divided into 5 groups (15 animals each group). The grouping and intraperitoneal injection schemes are as follows. (1) Uninfected DMSO group: each mouse was intraperitoneally injected with 100 μl of DMSO (1:1000 dilutions with corn oil); (2) DAS group: each mouse was intraperitoneally injected with 100 μl of DAS dissolved in DMSO (1:1000 dilutions with corn oil); (3) Y27632 group: each mouse was intraperitoneally injected with 100 μl of Y27632 dissolved in DMSO (1:1000 dilutions with corn oil); (4) DAS + Y27632 group: each mouse was intraperitoneally injected with 50 μl of DAS and 50 μl of Y27632; (5) Infected DMSO group: each mouse was intraperitoneally injected with 100 μl of DMSO (1:1000 dilutions with corn oil).

DAS and Y27632 were administered intraperitoneally three times (on days 0, 2 and 4). Except for the uninfected DMSO group, all other groups were infected orally with 200 *T. spiralis* muscle larvae (ML) per mouse on the second day after injection [5]. At 5 days post-infection (dpi), 15 mice from each group were sacrificed, and intestinal adults were recovered and worm burden was counted (n = 10). At 4 h after oral administration of 4 kDa FITC-dextran (FD-4; 100 μl, 5 mg) to each mouse, plasma was collected to measure intestinal permeability and intestinal tissues were collected (n = 5). qPCR and Western blot were used to ascertain the expression of CK8, RhoA, ROCK1, TJs, and inflammatory cytokines (TGF-β, IL-10, IL-1β and TNF-α) (IL-10 and TNF-α: BD Biosciences Pharmingen, USA; TGF-β and IL-1β: Dakewe, Beijing, China). The duodenum was also collected, fixed with 4% paraformaldehyde, embedded in paraffin, sectioned and stained with hematoxylin and eosin (HE) stain and periodic acid-Schiff reagent (PAS; Baso, Zhuhai, China) [54,55]. Pathological changes in intestinal mucosa of infected mice from each group were observed under a microscope, and the width of intestinal villi and the number of goblet cells per field (400×) were examined and counted [34,37].

### Statistical analysis

Data were analyzed using SPSS 26.0 and GraphPad Prism software. Results are expressed as the mean ± standard deviation (SD). The normality and homogeneity of the datum variance were evaluated by using the Shapiro-Wilk test and Levene’s test, respectively. Statistical analysis was performed using one-way analysis of variance (Pairwise comparisons across all groups were performed using Tukey’s honestly significant difference post-hoc test) and *t*-tests. A *P* value less than 0.05 was considered statistically significant.

## Results

### Molecular docking simulation of the binding between TsSPc and CK8

**Fig 1:**
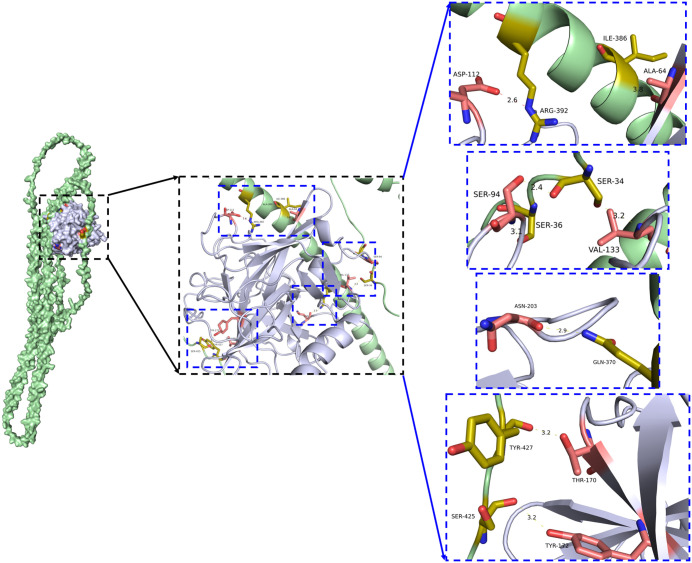
Molecular-docking simulation of the TsSPc–CK8 complex in Caco-2 cells. CK8 is depicted in green cartoon mode with residues shown as yellow sticks, while TsSPc is displayed in gray cartoon mode with residues shown as pink sticks. Hydrogen-bond distances are indicated by yellow dashed lines, and salt-bridge interactions are indicated by red dashed lines.

Molecular-docking results indicated that the binding free energy (Δ*iG*) between TsSPc and CK8 is –16.5 kcal/mol, signifying strong affinity. To further elucidate the interaction mechanism, we analyzed the binding mode of TsSPc and CK8. Fig 1 presents the docking model, in which TsSPc (gray cartoon) forms hydrogen bonds and salt bridges with amino-acid residues located near the interface of CK8 (green cartoon). Detailed distances for these hydrogen bonds and salt bridges are shown in S2 Table. Six hydrogen bonds and one salt bridge were identified between amino acid residues located at the TsSPc-CK8 interaction interface. The docking results confirmed that TsSPc and CK8 could stably be associated.

### Co-localization of rTsSPc with CK8 and their specific binding in Caco-2 cells

**Fig 2:**
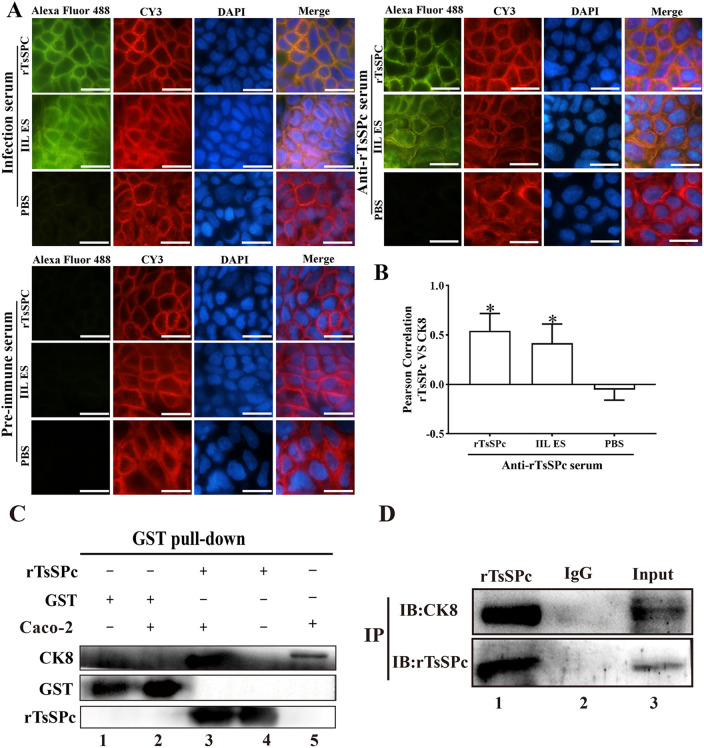
IFA, GST pull-down and Co-IP assay of specific binding between rTsSPc and CK8. Caco-2 cells were first incubated with rTsSPc, IIL ES antigens and PBS, followed by primary antibodies (anti-rTsSPc serum, infection serum, pre-immune serum, and anti-CK8 antibody). The secondary antibodies were Alexa Fluor 488-conjugated goat anti-mouse IgG and CY3-conjugated goat anti-rabbit IgG. DAPI was used to stain the nuclei blue. **A**: The localization of rTsSPc binding with CK8 was primarily in the intercellular junction of Caco-2 monolayer. Scale bar: 5 μm. **B**: Quantitative co-localization analysis of rTsSPc with CK8 in Caco-2 cells was performed by using ImageJ-produced Pearson’s correlation coefficients for each group, rTsSPc group exhibited a significantly higher Pearson’s coefficient, verifying that rTsSPc recognized by anti-rTsSPc serum was co-localized with CK8 among Caco-2 cells, *indicates *P* < 0.05 compared with the pre-immune serum group. **C**: GST pull-down assay. Lane 1: GSH agarose gel binds to GST; Lane 2: GSH agarose gel binds to GST and Caco-2 cell proteins; Lane 3: GSH agarose gel binds to rTsSPc and Caco-2 cell proteins; Lane 4: GSH agarose gel binds to rTSPc; Lane 5: Caco-2 cell proteins. **D:** Co-IP assay. Lane 1: Co-IP complex (rTsSPc and CK8 from Caco-2 cell proteins); Lane 2: normal mouse IgG; Lane 3: Caco-2 cellular proteins after being co-cultured with rTsSPc.

The IFA results showed that after Caco-2 cell monolayer was treated with rTsSPc for 2 h, incubation with anti-rTsSPc antibody and anti-mouse IgG-Alexa Fluor 488 conjugate showed green fluorescence signals around the cells. Meanwhile, natural CK8 protein in Caco-2 cells was recognized by anti-CK8 antibody and stained red fluorescence signal by CY3-labeled antibody. The yellow fluorescence observed in the rTsSPc-treated group, which is the integration of green and red fluorescence signals, indicated the co-localization of rTsSPc and CK8 in the intercellular space (Fig 2A). The quantitative co-localization analysis of rTsSPc with CK8 in Caco-2 cells has been performed by using ImageJ-produced Pearson correlation coefficients for each group. The results showed that compared with the PBS group, rTsSPc group exhibited a significantly higher Pearson coefficient (*F* = 11.11, *P* < 0.05) (Fig 2B), further verifying that rTsSPc recognized by anti-rTsSPc antibodies was co-localized with CK8 among Caco-2 cells. The results showed that rTsSPc and CK8 were co-localized in intercellular junction area, suggesting that rTsSPc was capable of specifically binding and interacting with CK8.

As shown in Fig 2C, the results of the GST pull-down assay indicated that the GST tag protein in lane 2 did not bind to Caco-2 cellular proteins, suggesting that there is no specific interaction between CK8 in Caco-2 cells and GST, thereby eliminating the potential interference of the GST tag protein. In lane 3, after Caco-2 cell soluble proteins were co-incubated with rTsSPc purified by GST affinity resin, Western blot showed that rTsSPc was bound to CK8, a Caco-2 cell receptor protein. This finding confirmed the specific binding between rTsSPc and CK8 receptor in Caco-2 cells.

The Co-IP results revealed that the protein complex produced from CK8 and rTsSPc with His tag could be effectively precipitated by agarose beads conjugated with anti-His antibody. But normal murine IgG could not capture CK8 or rTsSPc, indicating that there is indeed a specific interaction between CK8 and rTsSPc, resulting in the complex formation of CK8 and rTsSPc (Fig 2D). These results demonstrated that there is a specific binding and interaction between rTsSPc and the CK8 receptor in Caco-2 cells.

### Binding of rTsSPc to CK8 activated the RhoA/ROCK1 pathway

**Fig 3:**
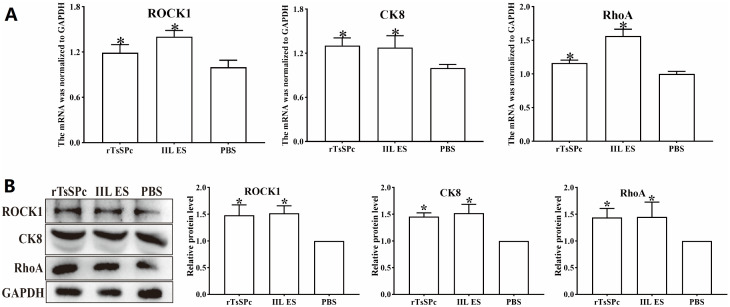
qPCR and Western blotting of activation of CK8 receptor and RhoA/ROCK1 pathway proteins in Caco-2 cells treated with rTsSPc and IIL ES antigens. Caco-2 cells were incubated with rTsSPc (20 μg/ml) for 2 h, and the mRNA and protein were extracted from the cells. The transcription and expression levels of CK8, RhoA, and ROCK1 were ascertained by qPCR (**A**) and Western blot (**B**), with GAPDH as the reference gene. * indicates a statistical difference compared to the PBS group, with *P* < 0.05.

As shown in Fig 3A, the CK8 transcription level in Caco-2 cells was significantly increased after stimulation with rTsSPc compared to the PBS control group (*F* = 12.43, *P* < 0.01). Western blot indicated that CK8 protein expression level in Caco-2 cells was significantly increased after stimulation with rTsSPc, compared to the PBS control group (*F* = 20.042, *P* < 0.01) (Fig 3B). RhoA was activated in the rTsSPc groups, with a significant increase in transcription levels (*F* = 57.579, *P* < 0.05); and a significant increase in its protein expression levels (*F* = 40.179, *P* < 0.01). The ROCK1 mRNA expression level was significantly increased (*F* = 16.519, *P* < 0.05). ROCK1 protein expression level was also significantly increased (*F* = 24.568, *P* < 0.01). These findings demonstrated that stimulation of Caco-2 cells with rTsSPc activated the CK8/RhoA/ROCK1 signaling pathway at their transcriptional and expressional level.

### Knockdown of CK8

**Fig 4:**
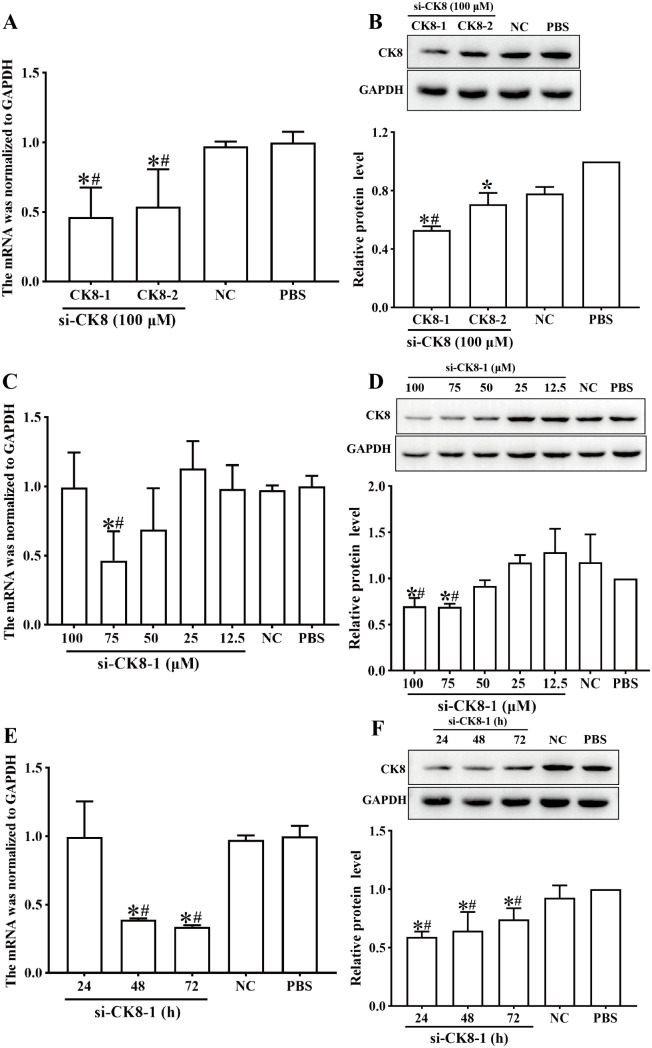
Transcription and expression levels of CK8 in Caco-2 cells after CK8 gene was knocked down. **A** and **B**: mRNA and protein expression levels of CK8 in Caco-2 cells transfected with different kinds of si-CK8. **C** and **D**: CK8 mRNA and protein expression levels in Caco-2 cells transfected with different doses of si-CK8-1. **E** and **F**: CK8 mRNA and protein expression levels at 24-72 h after transfection with 75 μM si-CK8-1. *P < 0.05 compared to the PBS group, ^#^*P* < 0.05 compared to the NC group.

After treatment with 100 μM si-CK8–1 and si-CK8–2 for 72 h, the CK8 mRNA expression levels were 0.464 and 0.539 times of the PBS group, respectively (*F* = 7.70, *P*_si-CK8–1_ < 0.01, *P*_si-CK8–2_ *<* 0.05), and the differences among the si-CK8–1, si-CK8–2, normal control (NC) group were significant (*P*_si-CK8–1_ < 0.01, *P*_si-CK8–2_ < 0.05); there was no significant difference between the PBS group and the NC group (*P* > 0.05) (Fig 4A). The CK8 protein expression levels in the si-CK8–1 and si-CK8–2 groups were 0.531 and 0.706 times of the PBS group, respectively (*F* = 51.299, *P*_si-CK8–1_ < 0.001, *P*_si-CK8–2_ < 0.001). The differences among the si-CK8–1 and NC group were statistically significant (*P <* 0.001), while there was no significant difference between the si-CK8–2 and NC group (*P* > 0.05) (Fig 4B), indicating that si-CK8–1 has a specific knockdown effect on CK8. Therefore, si-CK8–1 was used for the following experiments. In Caco-2 cells treated with 75 μM si-CK8–1, the CK8 mRNA expression level was significantly lower than the PBS and NC group, it was 0.464 times that of the PBS group (*F* = 4.042, *P*_PBS_ < 0.01, *P*_NC_ *<* 0.01); the CK8 protein expression level of the 75 μM si-CK8–1 group was 0.673 times that of the PBS group (*F* = 9.977, *P* < 0.05) (Fig 4C, 4D), but there was no significant difference in CK8 expression level between the NC and PBS group (*P* > 0.05). Hence, the subsequent transfection experiment was performed using 75 μM si-CK8–1. After transfection with 75 μM si-CK8–1 for 48 h, the CK8 mRNA transcription level was 0.389 times that of the PBS group (*F* = 18.543, *P* < 0.001), and the CK8 protein expression level was 0.645 times that of the PBS group (*F* = 9.949, *P* < 0.001) (Fig 4E, 4F), but there was no significant change of CK8 expression level in the NC group (*P* > 0.05). Therefore, transfection of Caco-2 cells with 75 μM si-CK8–1 and cultivation for 48 h after transfection are the optimal conditions for effectively silencing the CK8 gene.

### CK8 knockdown inhibited the rTsSPc-activated pathway and reduced TJs expression

**Fig 5:**
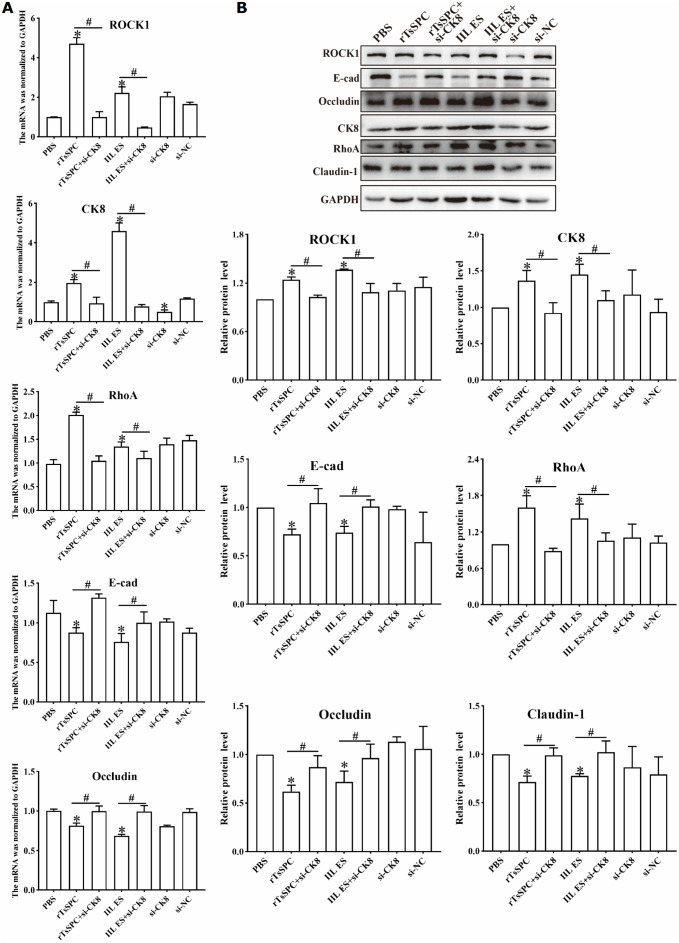
qPCR and Western blotting of CK8 knockdown blocking the rTsSPc-activated pathway and -reduced TJs expression. Caco-2 cells were incubated with rTsSPc (20 μg/ml) for 2 h after CK8 knockdown. Cellular mRNA and proteins were extracted, and the transcription and expression levels of CK8, RhoA, ROCK1 and TJs (E-cad, Occludin and Claudin-1) were assessed by qPCR (**A**) and Western blot (**B**), the GAPDH was used as the reference gene. *P < 0.05 compared to the PBS group, ^#^*P* < 0.05 compared between the two groups.

The qPCR results indicated that compared to the PBS group, the CK8 transcription level in rTsSPc-stimulated Caco-2 cells was obviously up-regulated (*F* = 182.3, *P* < 0.001). Similarly, the transcription levels of the pathway proteins RhoA and ROCK1 were also up-regulated significantly (*F*_RhoA_ = 28.88, *P* < 0.001; *F*_ROCK1 _= 269.00, *P* < 0.001). In contrast, the transcription levels of the TJs E-cad and Occludin were significantly down-regulated (*F*_E-cad _= 15.15, *P* < 0.05; *F*_Occludin _= 25.15, *P* < 0.001). In Caco-2 cells with CK8 knockdown after rTsSPc stimulation, the transcription levels of CK8, RhoA, and ROCK1 were significantly lower compared to the non-knockdown group (*F*_CK8_ = 5.079, *P* < 0.01; *F*_RhoA _= 14.000, *P* < 0.001; *F*_ROCK1 _= 36.330, *P* < 0.001). Meanwhile, the transcription levels of E-cad and Occludin were significantly increased (*F*_E-cad_ = 12.22, *P* < 0.001; *F*_Occludin _= 4.39, *P* < 0.05) (Fig 5A). However, after rTsSPc stimulation and CK8 knockdown, the transcriptional level of Claudin-1 mRNA in Caco-2 cells had no significant changes (*P* >* *0.05) (S1 Fig).

Western blot showed that after Caco-2 cells were treated with rTsSPc, the CK8 protein expression level was significantly up-regulated compared to the PBS group (*F* = 4.111, *P* < 0.05). The expression levels of ROCK1 and RhoA were also significantly higher than the PBS group (*F*_ROCK1_ = 9.477, *P* < 0.01; *F*_RhoA _= 8.116, *P* < 0.001). The expression levels of E-cad, Occludin, and Claudin-1 were significantly down-regulated compared to the PBS group (*F*_E-cad_ = 4.612, *P* < 0.05; *F*_Occludin_ = 6.719, *P* < 0.01; *F*_Claudin-1 _= 3.093, *P* < 0.05), indicating activation of the RhoA/ROCK1 pathway. In Caco-2 cells with CK8 knockdown, the protein expression levels of CK8, ROCK1 and RhoA in the rTsSPc group were significantly lower than the non-knockdown group (*F*_CK8_ = 4.111, *P* < 0.01; *F*_ROCK1_ = 9.477, *P* < 0.01; *F*_RhoA_ = 8.116, *P* < 0.001). The protein expression levels of E-cad, Occludin and Claudin-1 in the rTsSPc group after CK8 knockdown were significantly higher than the non-knockdown group (*F*_E-cad_ = 4.612, *P* < 0.05; *F*_Occludin_ = 6.719, *P* < 0.05; *F*_Claudin-1_ = 3.093, *P* < 0.05) (Fig 5B). These findings demonstrated that CK8 knockdown abolished the rTsSPc-up-regulated expression of the pathway proteins RhoA and ROCK1, restored and increased the rTsSPc-down-regulated expression levels of the TJs (E-cad, Occludin and Claudin-1).

### ROCK1 pathway inhibitor Y27632 suppressed the rTsSPc-activated pathway and restored TJs expression

**Fig 6:**
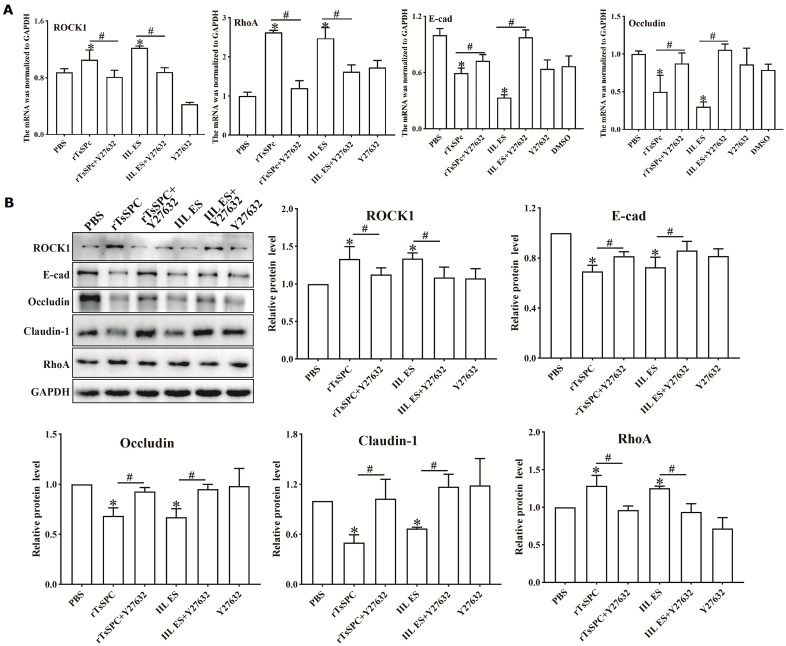
The ROCK1 pathway inhibitor Y27632 prevented rTsSPc from activating the ROCK1 pathway and reducing TJs expression. After Caco-2 cells were pre-treated with Y27632 (10 μM) for 24 h and then incubated with rTsSPc (20 μg/ml) for 2 h, cellular mRNA and proteins were extracted and the transcription and expression levels of RhoA, ROCK1 and TJs proteins E-cad, Occludin and Claudin-1 were ascertained by qPCR (**A**) and Western blot **(B)**, and GAPDH was used as the reference gene. * indicates *P* < 0.05 compared to the PBS group. ^#^ indicates *P* < 0.05 compared between the two groups.

qPCR results indicate that, compared to the PBS group, the transcription levels of RhoA and ROCK1 in rTsSPc-incubated Caco-2 cells were significantly up-regulated (RhoA: *F* = 41.927, *P* < 0.001; ROCK1: *F* = 94.000, *P* < 0.05); while the transcription levels of E-cad and Occludin were significantly down-regulated (E-cad: *F* = 42.15, *P* < 0.001; Occludin: *F* = 24.70, *P* < 0.05). However, After treatment with the ROCK1 pathway inhibitor Y27632, the transcription levels of RhoA and ROCK1 in Caco-2 cells were significantly lower than the non-inhibitor group (RhoA: *t* = 12.49, *P* < 0.001; ROCK1: *t* = 5.910, *P* < 0.01); while the transcrip*t*ion levels of E-cad and Occludin were significantly higher *t*han the non-inhibitor group (E-cad: *t* = 2.994, *P* < 0.05; Occludin: *t* = 3.211, *P* < 0.05) (Fig 6A).

Western blots showed that after rTsSPc treatment, the protein expression levels of ROCK1 and RhoA in Caco-2 cells were significantly up-regulated compared to the PBS group (ROCK1: *F* = 6.781, *P* < 0.01; RhoA: *F* = 14.618, *P* < 0.01). The TJs protein expression levels were significantly down-regulated compared to the PBS group (E-cad: *F* = 11.362, *P* < 0.001; Occludin: *F* = 8.403, *P* < 0.001; Claudin-1: *F* = 7.508, *P* < 0.01) (Fig 6B). The results indicated that rTsSPc activated the RhoA/ROCK1 pathway, decreased the TJs expression and disrupted enteral epithelial integrity. However, after treatment with the ROCK1 pathway inhibitor Y27632, the expression levels of ROCK1 and RhoA in the rTsSPc groups were significantly reduced compared to the non-inhibitor group (ROCK1: *t* = 2.980, *P* < 0.05; RhoA: *t* = 3.739, *P* < 0.05). The expression levels of the TJs (E-cad, Occludin, and Claudin-1) in the inhibitor-*t*reated rTsSPc groups were significantly higher than the non-inhibitor groups (E-cad: *t* = 3.500, *P* < 0.05; Occludin: *t* = 4.682, *P* < 0.01; Claudin-1: *t* = 3.681, *P* < 0.05). These results demonstrate tha*t* the ROCK1 pathway inhibitor Y27632 blocked the ROCK1 pathway activation by rTsSPc, restored and increased the expression levels of rTsSPc-reduced TJs proteins (E-cad, Occludin and Claudin-1).

### CK8 knockdown and RhoA/ROCK1 inhibitors abolished the rTsSPc-decreased TJs expression

**Fig 7:**
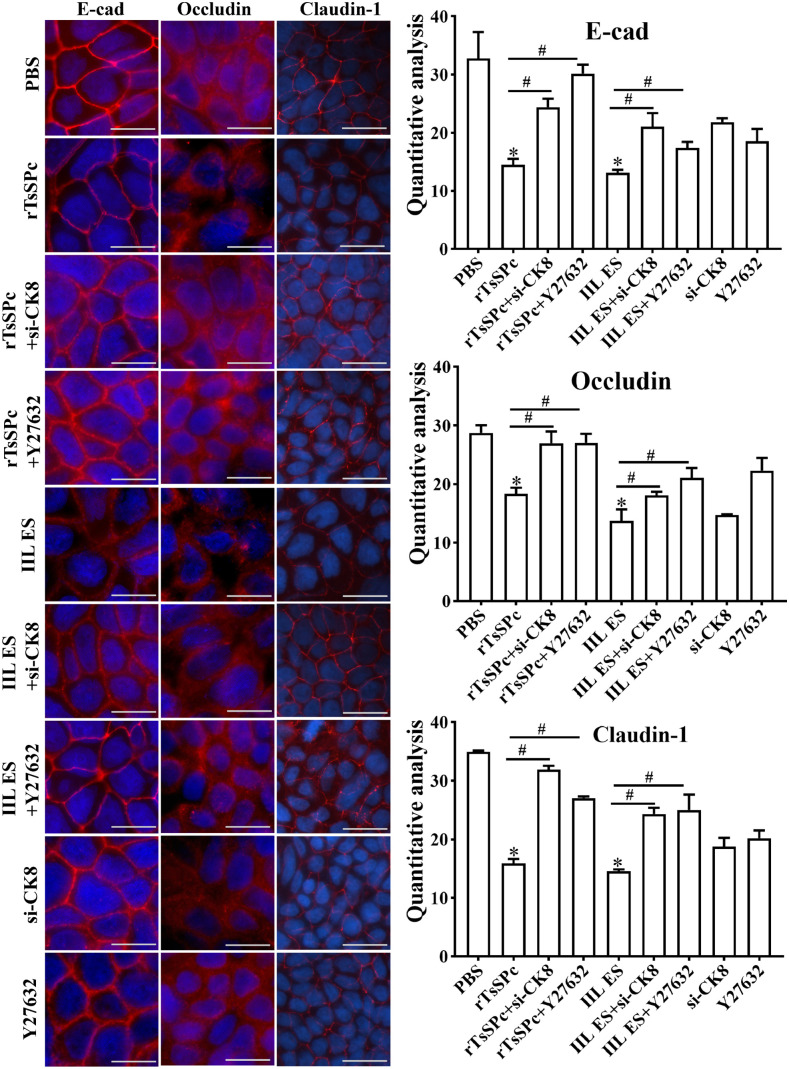
CK8 knockdown and ROCK1 inhibitor abolished rTsSPc-reduced TJs expression in Caco-2 monolayers by IFA. Caco-2 cells were incubated with rTsSPc after CK8 receptor knockdown and pre-treatment with ROCK1 inhibitor Y27632. The primary antibodies against E-cad, Occludin and Claudin-1 were used. The secondary antibody was CY3-conjugated goat anti-rabbit IgG (1:100). DAPI was used to stain the nuclei blue. Scale bar: 5 μm. *P < 0.05 compared to the PBS group. ^#^*P* < 0.05 compared between the two groups.

IFA results showed the fluorescence intensity of E-cad, Occludin and Claudin-1 in the rTsSPc-treated group was significantly lower than the PBS group (*F*_E-cad_ = 30.349, *P* < 0.001; *F*_Occludin _= 36.176, *P* < 0.001; *F*_Claudin-1_ = 127.825, *P* < 0.001). In the CK8 knockdown+rTsSPc group, the fluorescence intensity of E-cad, Occludin and Claudin-1 was significantly higher than the only rTsSPc group (*t*_E-cad _= 9.594, *P* < 0.001; *t*_Occludin _= 6.600, *P* < 0.01; *t*_Claudin-1_ = 28.16, *P* < 0.001). Similarly, in *t*he ROCK1 inhibitor Y27632 + rTsSPc group, *t*he fluorescence intensi*t*y of E-cad, Occludin and Claudin-1 was also significantly higher than the rTsSPc alone group (*t*_E-cad _= 14.50, *P* < 0.001; *t*_Occludin _= 8.186, *P* < 0.01; *t*_Claudin-1_ = 23.99, *P* < 0.001) (Fig 7). These results indicated that rTsSPc decreased the expression levels of TJs pro*t*eins (E-cad, Occludin and Claudin-1) in Caco-2 cells; whereas CK8 knockdown and ROCK1 inhibitor Y27632 abolished and increased rTsSPc-decreased TJs expression, therefore relieved the rTsSPc-damaged gut epithelial integrity.

### CK8 knockdown and the ROCK1 inhibitor block rTsSPc from disrupting Caco-2 cell barrier and inhibited larval invasion

**Fig 8:**
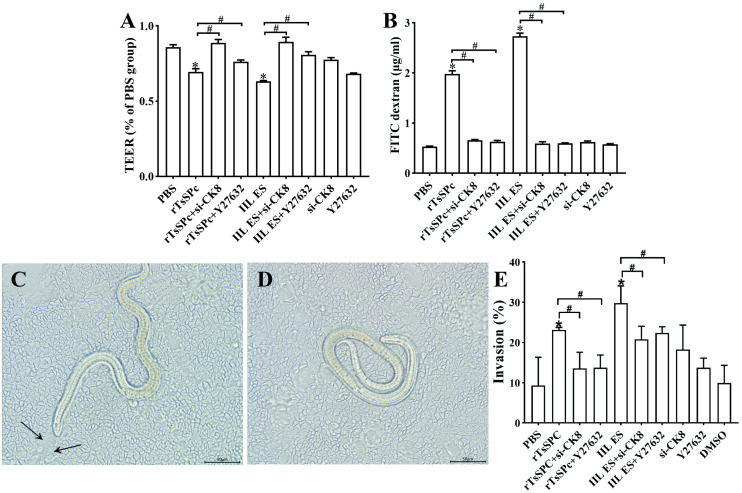
CK8 knockdown and ROCK1 inhibitor suppressed rTsSPc-induced disruption of the Caco-2 monolayer and blocked rTsSPc-promoted IIL invasion. **A:** Trans-epithelial electrical resistance (TEER). When Caco-2 cells reached full confluence, the cell monolayer integrity was assessed by measuring TEER. **B:** Paracellular permeability. FD-4 was added after the cells were incubated with rTsSPc for 2 h, the FD-4 transport from the upper chamber to the lower chamber across the cell monolayer in the Trans-well system was measured. **C:** IIL invading Caco-2 cells and disrupting the Caco-2 monolayer, arrows show the larval migrating track. **D:** Non-invaded and spiral IIL on the surface of the Caco-2 cell monolayer. **E:** CK8 knockdown and Y27632 blocked the rTsSPc promotion on IIL invasion of Caco-2 monolayer. Scale bar: 50 μm. The data represent the mean ± SD of three independent experiments. * indicates *P* < 0.05 compared to the PBS group; ^#^ indicates *P* < 0.05 compared between the two groups.

When Caco-2 cells reached full confluence, CK8 in the cells was first knocked down or the cells were pre-treated with the ROCK1 inhibitor Y27632, and then co-incubated with 20 μg/ml rTsSPc for 2 h. The TEER and FD4 permeability were measured. The results showed that, compared with the PBS group, rTsSPc treatment led to a significant decrease of the TEER (*F* = 79.067, *P* < 0.001) (Fig 8A) and a significant increase in FD4 flux across the Caco-2 monolayer (*F* = 1264.15, *P* < 0.001) (Fig 8B), indicating that rTsSPc significantly increased paracellular permeability, thereby disrupted the integrity of the Caco-2 monolayer barrier. But when the cells were pre-treated with si-CK8 and Y27632 and then incubated with rTsSPc, TEER was significantly increased relative to single rTsSPc group (*t*_rTsSPc+si-CK8_ = 10.73, *P* < 0.01; *t*_rTsSPc+Y27632_ = 4.786, *P* < 0.01); and a FD4 flux was evidently decreased (*t*_rTsSPc+si-CK8_ = 36.32, *P* < 0.001; *t*_rTsSPc+Y27632_ = 35.51, *P* < 0.001). The results suggested that both CK8 knockdown and ROCK1 inhibitor Y27632 abolished and decreased the rTsSPc-increased paracellular permeability.

The results of the *in vitro* larval invasion test showed that the invaded larvae were active and migrating in Caco-2 monolayer (Fig 8C), but the non-invaded larvae were still suspended in culture medium and spirally coiled (Fig 8D). rTsSPc clearly promoted the IIL invasion of Caco-2 monolayer, with a larval invasion rate of 23.16% which were significantly higher than 9.34% of the PBS group (*χ²* = 79.062, *P* < 0.001). After CK8 knockdown and inhibitor Y27632 were used, larval invasion of the rTsSPc + si-CK8 and rTsSPc + Y27632 group was 13.59 and 13.75%, respectively, and they were reduced by 9.57% and 9.40% compared to the single rTsSPc group (*χ²*_si-CK8 _= 8.727, *P* < 0.01; *χ²*_Y27632 _= 8.727, *P* < 0.01) (Fig 8E). These results suggested that CK8 knockdown and the ROCK1 pathway inhibitor Y27632 abolished and eliminated the rTsSPc facilitation on the IIL invasion into Caco-2 cells, and demonstrated that the CK8 receptor and the ROCK1 pathway played the important roles during *T. spiralis* larval invasion of intestinal mucosa.

### DAS and Y27632 decreased intestinal permeability and reduced intestinal adult burden in infected mice

**Fig 9:**
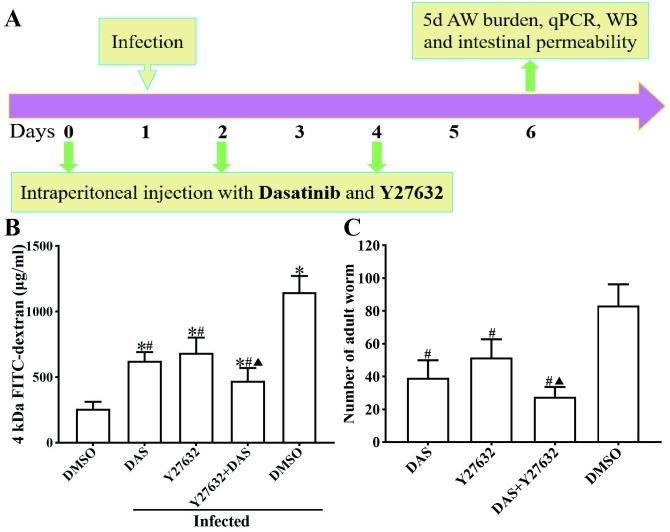
Intraperitoneal injection protocol for inhibitors (A), inhibitors DAS and Y27632 reduced intestinal permeability (B) and intestinal adult worm burden (C) of infected mice. **A:** Mice were intraperitoneally injected according to the above-described grouping. DAS and Y27632 were administered intraperitoneally three times (on days 0, 2, and 4). Except for the uninfected DMSO group, all other groups of mice were orally infected with 200 *T. spiralis* muscle larvae (ML) per mouse on the second day after injection. **B:** DAS and Y27632 decreased the blood plasma FD-4 content increase caused by *T. spiralis* infection. Each group had five replicates. **C:** DAS and Y27632 reduced intestinal adult worm burden at 5 dpi. Data are presented as mean ± SD (n = 10). * *P* < 0.05 compared with the uninfected DMSO group; ^#^*P* < 0.05 compared with the infected DMSO group; ^▲^*P* < 0.05 compared with the only DAS or Y27632 group.

To determine whether CK8 inhibitor DAS and ROCK1 inhibitor Y27632 reduce intestinal permeability in infected mice, mice were intraperitoneally injected with DAS, Y27632 or DAS + Y27632, respectively (Fig 9A). FD-4 was then administered by gavage, and plasma was collected to measure the FD-4 content in each group of mice. The results showed that DAS and Y27632 effectively alleviated intestinal permeability increase caused by *T. spiralis* infection (Fig 9B). Compared with the uninfected DMSO group, the FD-4 content in the infected DMSO group was increased by 4.43 times (*F* = 71.151, *P* < 0.001), further verifying that *T. spiralis* infection significantly increased intestinal permeability. After intraperitoneal injection with inhibitor DAS or Y27632, intestinal permeability in the DAS, Y27632 and DAS + Y27632 groups was significantly reduced, compared with the infected DMSO group. The FD-4 content in the DAS, Y27632 and DAS + Y27632 group was decreased by 1.84 times (*t* = 9.157, *P* < 0.001), 1.68 *t*imes (*t* = 6.693, *P* < 0.001), and 2.44 times (*t* = 10.53, *P* < 0.001), respectively. Compared with the DAS group or Y27632 group alone, the FD-4 content in the DAS + Y27632 group was reduced by 1.45 times (*t* = 3.176, *P* < 0.01) and 1.32 times (*t* = 3.429, *P* < 0.01), respectively. The resul*t*s showed that *t*he CK8 receptor inhibitor DAS and the ROCK1 inhibitor Y27632 effectively abolished and reduced intestinal permeability increase resulted from *T. spiralis* infection, and significantly enhanced intestinal epithelium integrity and its barrier function. The results further suggested that DAS and Y27632 prevented the binding of TsSPc to intestinal epithelial receptor CK8 and inhibited the activation of the ROCK1 pathway, thereby up-regulated intestinal epithelial TJs protein expression, restored intestinal barrier integrity and impeded larval invasion.

The results of intestinal adult worm burdens showed that, compared with the DMSO group, the adult worm burden in the DAS, Y27632, and DAS + Y27632 group was reduced by 2.13 times (*t* = 8.336, *P* < 0.001), 1.62 times (*t* = 5.981, *P* < 0.001), and 3.02 *t*imes (*t* = 12.42, *P* < 0.001), respectively. The adult worm burden in the DAS + Y27632 group was reduced by 1.42 times (*t *= 2.991, *P* < 0.01) and 1.87 times (*t *= 5.986, *P* < 0.001) compared with the only DAS or Y27632 alone group (Fig 9C). The results indica*t*ed that the CK8 receptor antagonist DAS and the ROCK1 pathway inhibitor Y27632 effectively prevented larval invasion and development, thereby reduced intestinal adult worm burden. The inhibitory effect of DAS + Y27632 was superior to that of single DAS or Y27632 alone.

### DAS and Y27632 inhibited the increased transcription of CK8, RhoA and ROCK1, and restored the decreased TJs transcription in infected mice

**Fig 10:**
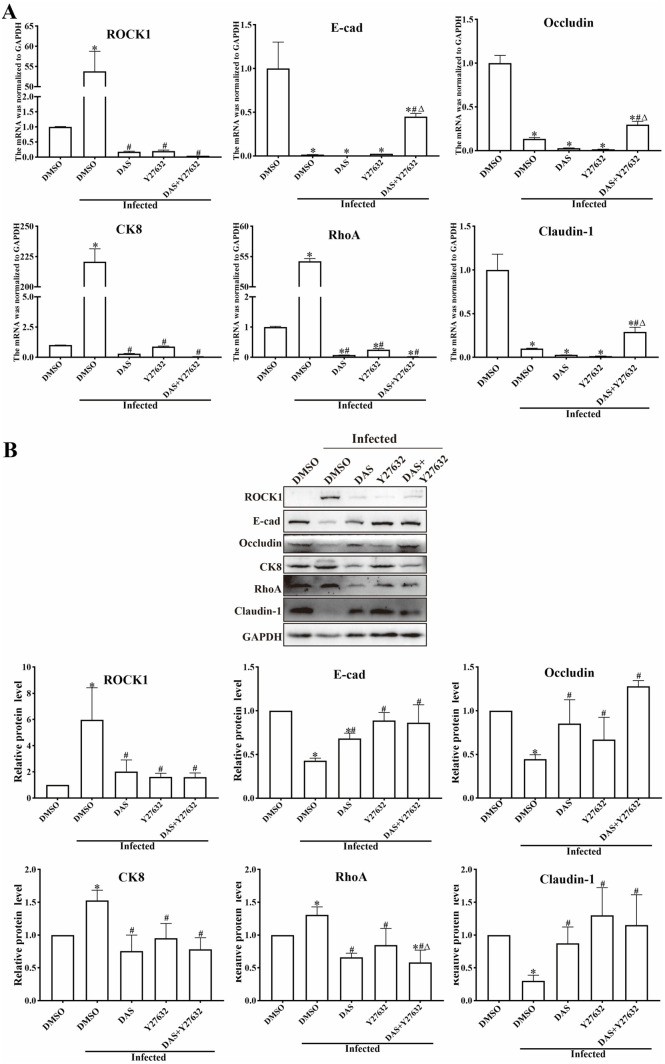
DAS and Y27632 down-regulated the mRNA and protein expression of CK8-RhoA/ROCK1 pathway and up-regulated TJs expression in intestines of infected mice. At 5 dpi, small intestines were collected from infected mice, mRNA and tissue proteins were extracted, and the mRNA and protein expression levels of CK8, RhoA, ROCK1, and tight junctions (TJs) in intestinal mucosa were analyzed by qPCR (**A**) and Western blot **(B)**. GAPDH was used as the reference gene. Each experiment was repeated three times. *P < 0.05 compared to the uninfected DMSO group; ^#^*P* < 0.05 compared to the infected DMSO group; ^△^*P* < 0.05 compared to the individual DAS or Y27632 alone group.

At 5 dpi, all infected mice were sacrificed, and small intestines were collected. mRNA and tissue proteins were extracted, and the transcription and expression levels of CK8, RhoA, ROCK1, and TJs in intestinal mucosal epithelium were analyzed by qPCR and Western blot. qPCR results showed that, compared with the uninfected group, transcription of CK8, RhoA and ROCK1 in the infected group was increased by 220.69, 54.21 and 53.79 times, respectively (*F*_CK8_ = 1261.45, *F*_RhoA_ = 42545.37, *F*_ROCK1_ = 344.48, *P* < 0.001) (Fig 10A). However, compared with the infected DMSO group, transcription of CK8, RhoA and ROCK1 in the DAS + Y27632 group was significantly reduced (*t*_CK8_ = 35.60, *t*_RhoA_ = 208.50, *t*_ROCK1_ = 18.67, *P* < 0.001). Compared with the uninfected DMSO group, the mRNA transcription levels of E-cad, Occludin and Claudin-1 in the infected DMSO group was decreased significantly (*F*_E-cad_ = 30.61, *F*_Occludin_ = 265.31, *F*_Claudin-1_ = 72.89, *P* < 0.001). Compared with the infected DMSO group, the transcription levels of E-cad, Occludin, and Claudin-1 in the DAS + Y27632 group was increased by 28.77, 2.23 and 2.91 times, respectively (*t*_E-cad_ = 20.69, *P* < 0.001; *t*_Occludin_ = 7.03, *P* < 0.01; *t*_Claudin-1_ = 6.201, *P* < 0.01). The results showed tha*t* the combina*t*ion of DAS and Y27632 partially counteracted the transcription level decreases of E-cad, Occludin and Claudin-1 caused by *T. spiralis* infection.

Western blot results showed that, compared with the uninfected group, expression level of CK8, RhoA, and ROCK1 in infected group was increased by 1.52-, 1.30-, and 5.97-fold, respectively (*F*_CK8_ = 8.674, *P* < 0.01; *F*_RhoA_ = 10.413, *P* < 0.05; *F*_ROCK1_ = 8.679, *P* < 0.001) (Fig 10B). Compared with the infected DMSO group, the expression levels of CK8, RhoA, and ROCK1 in the DAS + Y27632 group were significantly reduced (*t*_CK8_ = 5.436, *P* < 0.01; *t*_RhoA_ = 5.577, *P* < 0.01; *t*_ROCK1_ = 3.066, *P* < 0.05). Compared with the uninfected DMSO group, expression levels of E-cad, Occludin and Claudin-1 in infected DMSO group were significantly decreased (*F*_E-cad_ = 13.44, *P* < 0.001; *F*_Occludin_ = 10.27, *P* < 0.01; *F*_Claudin-1_ = 4.78, *P* < 0.05). Compared with the infected DMSO group, expression levels of E-cad, Occludin, and Claudin-1 in the DAS + Y27632 group were increased by 2.00-, 2.87-, and 3.81-fold, respectively (*t*_E-cad_ = 3.603, *P* < 0.05; *t*_Occludin_ = 17.56, *P* < 0.001; *t*_Claudin-1_ = 3.129, *P* < 0.05). These findings further indicated that while *T. spiralis* invaded gut mucosa, its secreted TsSPc protein bound to gut epithelial CK8 receptor and activated the RhoA/ROCK1 signaling pathway, reduced the expression of gut epithelial TJs and disrupted intestinal barrier integrity, as a result, mediated the larval invasion of gut mucosa.

### DAS and Y27632 alleviated intestinal inflammation in infected mice

**Fig 11:**
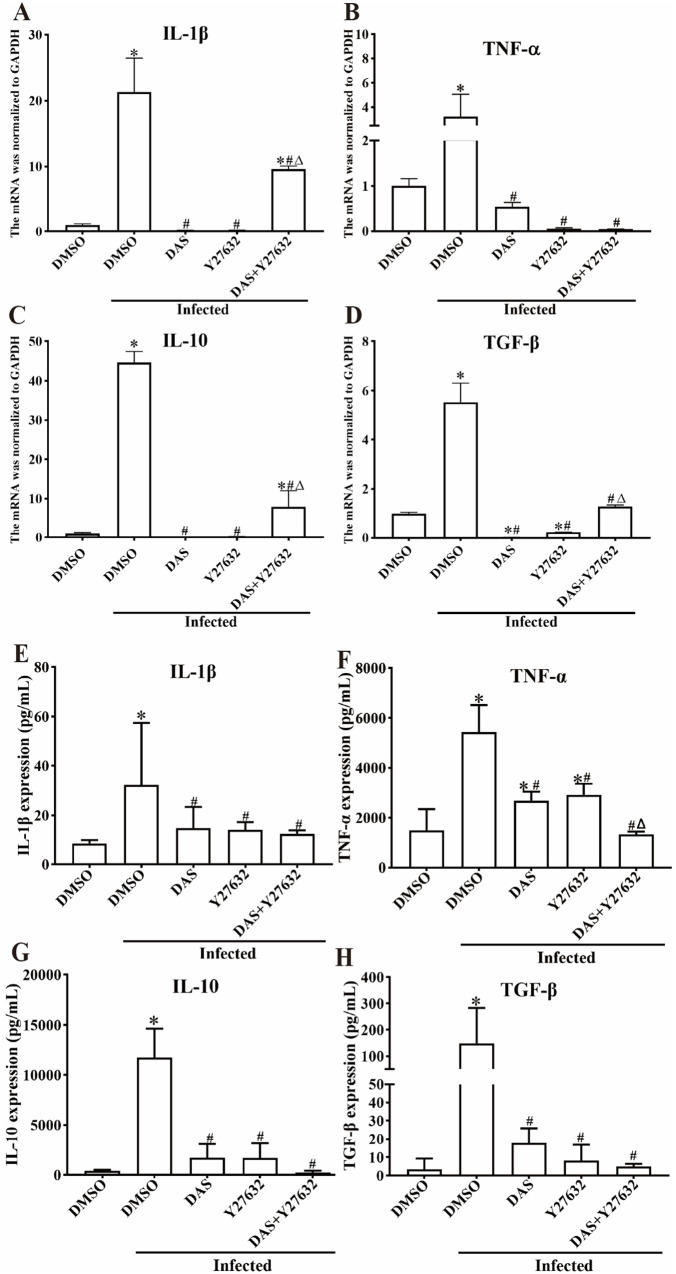
qPCR (A-D) and ELISA (E-H) detection of inflammatory cytokines in intestine of *T. spiralis*-infected mice. **A** and **B**: DAS and Y27632 inhibited the transcription up-regulation of pro-inflammatory cytokines (IL-1β and TNF-α). **C** and **D**: DAS and Y27632 inhibited the transcription up-regulation of anti-inflammatory cytokines (IL-10 and TGF-β). **E** and **F**: DAS and Y27632 reduced the increased expression levels of pro-inflammatory cytokines (IL-1β and TNF-α). **G** and **H**: DAS and Y27632 reduced the increased expression levels of anti-inflammatory cytokines (IL-10 and TGF-β). *P < 0.05 compared with the uninfected DMSO group; ^#^*P* < 0.05 compared with the infected DMSO group; ^△^*P* < 0.05 compared with only DAS or Y27632 alone group.

Expression levels of inflammatory cytokines in intestine of infected mice were assessed using qPCR and ELISA. qPCR results showed that compared with uninfected DMSO group, transcriptional levels of pro-inflammatory cytokines (IL-1β and TNF-α) and anti-inflammatory cytokines (IL-10 and TGF-β) in the infected DMSO group were significantly increased (*F*_IL-1β_ = 47.650, *F*_TNF-α_ = 7.913, *F*_TGF-β_ = 124.53, *F*_IL-10_ = 22469, *P* < 0.001) (Fig 11A-11D), suggesting that *T. spiralis* infection increased the transcription levels of inflammatory cytokines. After the inhibitors were administrated, compared with the infected DMSO group, the transcriptional levels of pro-inflammatory cytokines IL-1β and TNF-α in the DAS, Y27632 and DAS + Y27632 groups were significantly decreased (IL-1β: *P* < 0.001; TNF-α: *P* < 0.01), and the transcriptional levels of anti-inflammatory cytokines IL-10 and TGF-β in the DAS, Y27632 and DAS + Y27632 groups were also significantly decreased (IL-10: *P* < 0.001; TGF-β: *P* < 0.01). The ELISA results showed that compared with the uninfected DMSO group, the expression of pro-inflammatory cytokines (IL-1β and TNF-α) and anti-inflammatory cytokines (IL-10 and TGF-β) in the infected DMSO group were obviously increased (*F*_IL-1β_ = 2.887, *P* < 0.01; *F*_TNF-α_ = 30.282, *P* < 0.001; *F*_TGF-β_ = 4.391, *P* < 0.01; *F*_IL-10_ = 47.933, *P* < 0.001) (Fig 11E-11H). After inhibitors were administrated, compared with the infected DMSO group, the expression of pro-inflammatory cytokines IL-1β and TNF-α in the DAS, Y27632 and DAS + Y27632 groups were significantly decreased (IL-1β: *P* < 0.05; TNF-α: *P* < 0.001), and the expression levels of anti-inflammatory cytokines IL-10 and TGF-β in the DAS, Y27632, and DAS + Y27632 groups were also significantly decreased (IL-10: *P* < 0.001; TGF-β: *P* < 0.01). These results indicated that the combination of DAS and Y27632 inhibited *T. spiralis* invasion of intestinal mucosa, reduced the production of pro-inflammatory and anti-inflammatory cytokines, and thereby alleviated intestinal mucosal inflammatory response.

### DAS and Y27632 alleviate intestinal pathological changes in infected mice

**Fig 12:**
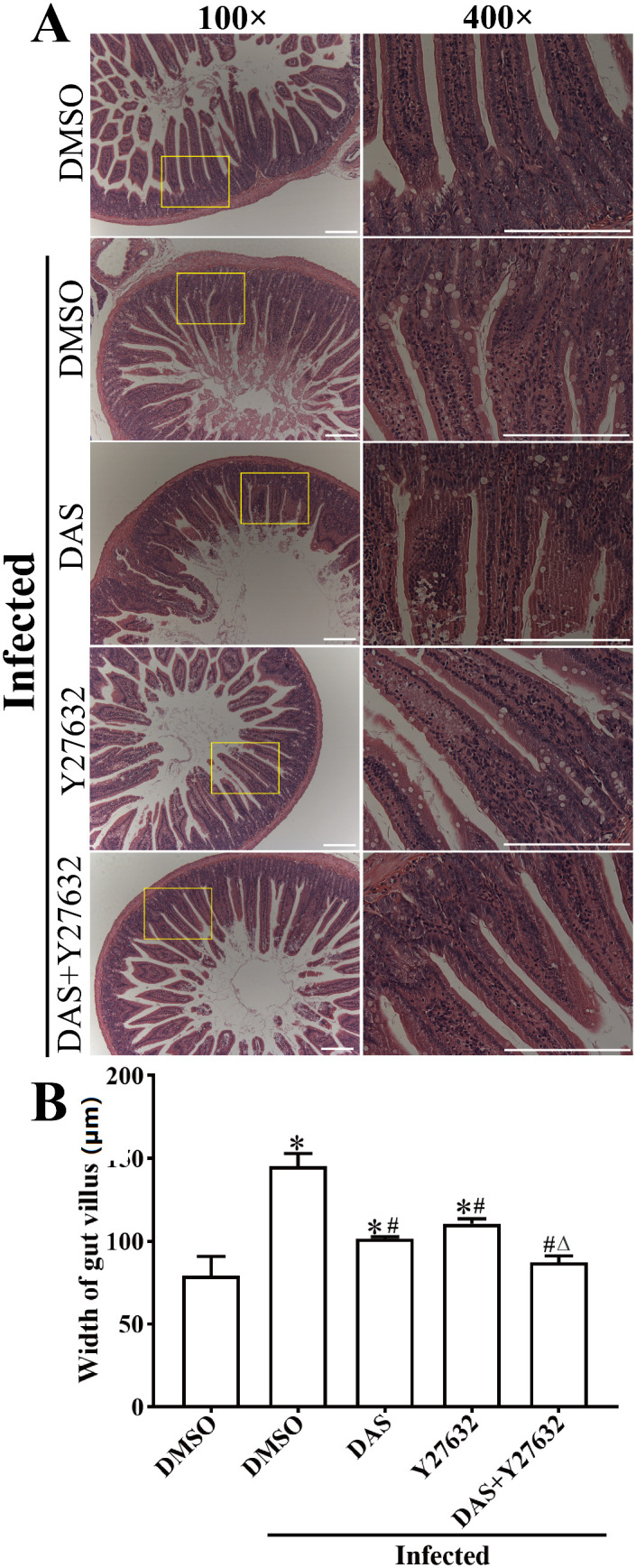
HE staining showed that DAS and Y27632 alleviated intestinal inflammation in *T. spiralis*-infected mice. **A**: HE staining of duodenal sections. Microscopic observation revealed that DAS and Y27632 relieved intestinal inflammation in infected mice. Scale bar = 200 μm. **B**: Intestinal villus width (n = 3). These figures indicated that both CK8 receptor inhibitor DAS and ROCK1 inhibitor Y27632 obviously ameliorated enteral inflammation in *T. spiralis*-infected mice. *P < 0.05 compared to the uninfected DMSO group; ^#^*P* < 0.05 compared to the infected DMSO group; ^△^*P* < 0.05 compared to the individual DAS or Y27632 alone group.

After intraperitoneal pre-injection of DAS, Y27632 or DAS + Y27632, mice were infected with 200 *T. spiralis* ML. At 5 dpi, mice were sacrificed, and duodenal tissues were collected to prepare tissue sections for HE and PAS staining. The HE staining showed that intestinal villi in the uninfected DMSO group appeared relatively normal, while those in the infected DMSO group exhibited the villous destruction and swelling. Compared to the uninfected DMSO group, intestinal villi in the infected DMSO group were significantly widened (*F* = 44.84, *P* < 0.0001). Compared to the infected DMSO group, intestinal villi of DAS, Y27632 and DAS + Y27632 group showed the significantly alleviated inflammation reaction (*P* < 0.001) (Fig 12A). Compared to the infected DMSO group, intestinal villous width in the DAS, Y27632 and DAS + Y27632 group was significantly reduced (*P* < 0.001); intestinal villous width in the DAS + Y27632 group was significantly smaller than the only DAS or Y27632 alone group (*P* < 0.05) (Fig 12B).

**Fig 13:**
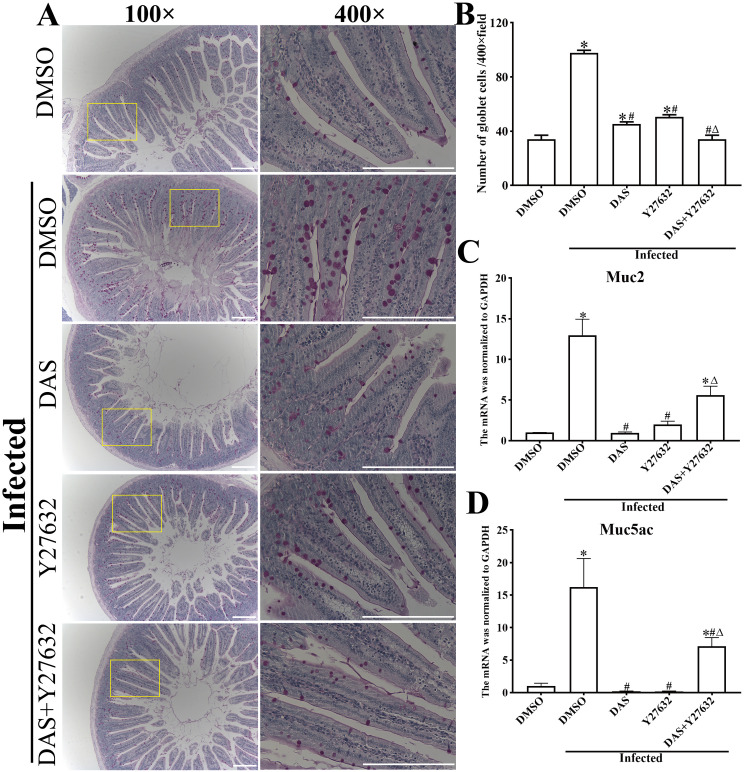
PAS staining of intestinal sections from *T. spiralis*-infected mice pretreated with DAS and Y27632. **A:** PAS staining of duodenal sections observed under a microscope. Compared with the infected DMSO group, the size and number of intestinal goblet cells in the DAS and Y27632 group was obviously reduced. Scale bar = 200 μm. **B**: Number of intestinal goblet cells (n = 3). **C**: Muc2 mRNA transcription level (n = 3). **D**: Muc5ac mRNA transcription level (n = 3). These figures indicated that both inhibitors DAS and Y27632 distinctly reduced the number of goblet cells and decreased expression levels of Muc2 and Muc5ac in gut mucosa of *T. spiralis*-infected mice. *P < 0.05 compared to the uninfected DMSO group; ^#^*P* < 0.05 compared to the infected DMSO group; ^△^*P* < 0.05 compared to the individual DAS or Y27632 alone group.

PAS staining revealed that, compared with the uninfected DMSO group, the number of goblet cells in the infected DMSO group were significantly increased (*F* = 291.35, *P* < 0.001) (Fig 13A). However, compared with the infected DMSO group, the number of goblet cells in the DAS, Y27632 and DAS + Y27632 group was significantly reduced (*P* < 0.001) (Fig 13B). qPCR results showed that, compared to the uninfected DMSO group, Muc2 and Muc5ac mRNA levels in the infected DMSO group were significantly increased (*F*_Muc2_ = 16.765, *P* < 0.001; *F*_Muc5ac_ = 34.15, *P* < 0.001). Compared to the infected DMSO group, Muc2 mRNA level in the DAS, Y27632 and DAS + Y27632 group was reduced by 17.01, 8.10, and 2.90 times, respectively (*P* < 0.001) (Fig 13C); Muc5ac mRNA level in the DAS, Y27632 and DAS + Y27632 group was reduced by 84.63, 91.16, and 2.28 times, respectively (*P* < 0.001) (Fig 13D).

The above results indicated that both of CK8 receptor inhibitor DAS and ROCK1 pathway inhibitor Y27632 relieved intestinal inflammation and decreased the expression of Muc2 and Muc5ac in intestinal mucosa of *T. spiralis-*infected mice.

## Discussions

*Trichinella spiralis* is an intestine- and tissue-dwelling zoonotic parasitic nematode which infects many kinds of mammal animals, and is the main pathogen of trichinellosis [1]. The adult worms and larvae of *T. spiralis* parasitize in small intestine and skeletal muscles of the same host, and larval invasion of intestinal mucosa is the pivotal step for *T. spiralis* infection [52]. The IIL primarily disrupt intestinal epithelial TJs proteins by their secreted proteases, thereby damaging intestinal epithelial integrity and barrier function [13,29]. At present, more than 80 kinds of serine proteases have been identified in the ES proteins of different *T. spiralis* developmental stage worms, with serine proteases accounting for the highest proportion among all protease classifications [56–58]. *T. spiralis* serine proteases play an important role in assisting the larval disruption of intestinal epithelial barrier and invasion of intestinal mucosa [12,59,60].

There are various methods for verifying protein-protein interactions, several common ones include the yeast two-hybrid system, GST pull-down, mass spectrometry and Co-IP, and protein microarray. In our previous studies, GST pull-down and mass spectrometry showed Caco-2 cell CK8 protein interacted with rTsSPc were identified [17]. Therefore, the objective of this study was to further verify whether TsSPc binds and interacts with CK8 receptor in intestinal epithelium cells, and whether the binding disrupts intestinal epithelial integrity and promotes larval invasion.

Molecular docking is a method that uses computer technology to assist in predicting the geometric conformation of two or more molecules after binding [61]. It can help explain the interaction mechanisms between protein and ligand, including the recognition of binding sites, conformational changes, and the evaluation of binding affinity [62]. In this study, CK8 was docked with rTsSPc, yielding an optimal score of –16.5 kcal/mol. The interaction is stabilized by hydrogen bonds and salt bridges. Previous reports indicate that Δ*iG* ≤ –5 kcal/mol is considered “binding,” Δ*iG* ≤ –7 kcal/mol is generally viewed as “strong binding,” and Δ*iG* ≤ –9 kcal/mol denotes “high-affinity” [63]; therefore, the TsSPc–CK8 interaction qualifies as the high-affinity. To further corroborate the reliability of the docking results, the co-localization of TsSPc–CK8 in Caco-2 cells was confirmed by subsequent IFA, GST pull-down, and Co-IP assays. The IFA colocalization is an effective method for detecting the spatial distribution and interactions of proteins and other biomolecules within cells [34]. Its principle involves using fluorescently labeled antibodies to specifically bind to target molecules, and observing the colocalization of these molecules among or within cells through a fluorescence microscope, thereby inferring their interactions or functional connections. We used IFA to observe whether rTsSPc colocalizes with CK8 in Caco-2 cells. The results showed that the yellow parts represent the colocalization sites of CK8 (red fluorescence) and rTsSPc (green fluorescence), which are localized on cell membrane. GST pull-down is one of the classic *in vitro* experimental techniques to study protein-protein interactions. Based on the high-affinity interaction between GST and glutathione (GSH), it is widely used to study protein interaction networks, including key proteins in signaling pathways, interaction networks of disease-related proteins, and screening of drug targets. In the present study, rTsSPc with GST tag was incubated with glutathione agarose beads (GSH beads) and then co-incubated with Caco-2 cells to verify whether CK8 in Caco-2 that interacts with rTsSPc is pulled down. Western blotting showed that rTsSPc interacted with CK8 in Caco-2 cells, but in the control groups with blank GSH beads and GST-purified protein, no interaction with CK8 was found, indicating that rTsSPc interacted specifically with CK8 *in vitro*. The Co-IP is a classic technique for studying protein interactions; it is based on the principle of specific antigen-antibody binding, and can verify protein interactions in their native cellular state [64]. Co-IP captures target proteins and their interacting protein complexes through specific antibodies, and then identifies the interacting proteins through Western blot or mass spectrometry [65]. In this study, Co-IP was also used to verify whether rTsSPc binds to CK8 in native Caco-2 cells, and the results showed that the anti-CK8 antibody could pull down the complex of rTsSPc binding with CK8, indicating that rTsSPc bound to CK8 in native Caco-2 cells.

CK8 is a type II cytokeratin and a member of the intermediate filament family, which is involved in maintaining the structural integrity and stability of epithelial cells. It commonly forms complexes with CK18 and is expressed in glandular epithelial cells, transitional epithelial cells, and hepatocytes in normal tissues. Previous studies indicated that Rho regulates the actin cytoskeleton through ROCK and LIM kinases [66]. It was discovered that CK8/18, by modulating the stability of the cytoskeleton, affects the activity of the RhoA/ROCK signaling pathway, thereby regulates cellular mechanical properties and interactions with the extracellular matrix [67]. The up-regulation of CK8 expression leads to decreased fluorescence intensity of intestinal epithelial TJs proteins and increased RhoA activity [21]. The rigidity of the actin cytoskeleton is associated with the activation of the ROCK signaling pathway by cytokeratin 8/18. The RhoA signaling pathway plays a pivotal role in the infection of parasites, such as *Plasmodium, Toxoplasma gondii*, *Cryptosporidium parvum* and *Theileria annulata* [68]. Based on these findings, we hypothesized that rTsSPc interacted with CK8 in Caco-2 cells, thereby activated the RhoA/ROCK1 signaling pathway, reduced expression levels of gut epithelial TJs proteins, disrupted intestinal barrier, and promoted *T. spiralis* larval invasion. Therefore, in this study, we focused on the CK8-mediated activation of the RhoA/ROCK1 pathway in the mechanism of *T. spiralis* invasion of gut mucosa. Experimental results showed that after stimulation of Caco-2 cells with rTsSPc, the expression levels of CK8 receptor and pathway proteins RhoA/ROCK1 were significantly increased; whereas the expression levels of TJs (E-cad, Occludin and Claudin-1) were obviously decreased. After CK8 in Caco-2 cells was knocked down, the expression levels of CK8, RhoA and ROCK1 were evidently decreased, while the expression levels of TJs (E-cad, Occludin and Claudin-1) were clearly restored and increased. Similarly, after Caco-2 cells were treated with ROCK inhibitor Y27632, the expression levels of RhoA and ROCK1 were decreased, while expression levels of TJs proteins were increased observably. Tight junctions are a special type of intercellular connection structure, mainly found in epithelial and endothelial cells, and they act the function as barriers and for cell adhesion [69]. Therefore, TJs proteins play a crucial role in maintaining intestinal epithelial integrity and barrier function. Our results demonstrated that the interaction of rTsSPc with CK8 in Caco-2 cells activated the RhoA/ROCK1 pathway, reduced the TJs expression, and damaged intestinal barrier function.

TEER is widely used for studying intestinal barrier, pulmonary barrier and blood-brain barrier. It assesses the TJs integrity among intestinal epithelial cells by measuring the electrical resistance across a cellular monolayer. The higher the resistance value, the stronger the cellular barrier function [70]. Caco-2 cells, which possess microvilli and resemble the enterocytes of small intestine in morphology and function, are internationally recognized as an *in vitro* model for studying tight junctions. Fluorescently labeled molecules (such as FITC-Dextran-4 kDa) were added to the apical side of the cellular monolayer, and their fluorescence intensity on the basal side can be detected to evaluate paracellular permeability. The higher the fluorescence intensity, the greater the enteral permeability [47]. In this study, the effect of rTsSPc binding and interacting with CK8 on intestinal permeability was assessed by measuring TEER and the fluorescence intensity of FD-4. After Caco-2 cells were co-incubated with rTsSPc, the TEER was decreased, and paracellular permeability was significantly increased. However, CK8 knockdown and pre-treatment with the ROCK inhibitor Y27632 in Caco-2 cells restored the TEER to normal levels and reduced paracellular permeability to normal levels. The results indicated that CK8 knockdown and ROCK inhibitor restored and increased the rTsSPc-decreased the TJs expression levels.

To evaluate the role of rTsSPc binding with CK8 receptor in larval invasion of intestinal epithelium, an *in vitro* invasion test was conducted. The results showed that rTsSPc significantly promoted larval invasion of Caco-2 monolayer. However, when CK8 in Caco-2 cells were knockdown or Caco-2 monolayer was pretreated with ROCK1 pathway inhibitor Y27632, the larval invasion was significantly inhibited compared to only rTsSPC-treated Caco-2 cells. Additionally, our previous study indicated that the blockade of TsSPc using anti-TsSPc antibodies and silencing of TsSPc gene by specific dsRNA obviously impeded the *in vitro* larval invasion. The findings further certified that rTsSPc compromised the integrity of intestinal epithelial barrier to facilitate larval invasion by binding and interacting with CK8 in Caco-2 cells. This rTsSPc promotion on larval invasion could be abolished by CK8 knockdown and ROCK1 inhibitor Y27632. These findings indicated the significance of the TsSPc-CK8 interaction mechanism in the TsSPc-mediated larval invasion, and suggested that TsSPc might be considered as a molecular target for anti-*Trichinella* vaccines [16].

To further investigate the effect of TsSPc binding with CK8 receptor on gut epithelial barrier *in vivo*, mice were intraperitoneally injected with CK8 inhibitor (DAS) and ROCK1 pathway inhibitor (Y27632). After pretreatment of mice with two inhibitors, intestinal AW burdens of infected mice at 5 dpi were significantly reduced, indicating that the inhibitors of CK8 receptor and ROCK1 pathway obviously suppressed *T. spiralis* larval invasion and development in host intestine, thereby evidently decreased intestinal adult worm burdens [9]. Intestinal permeability in infected mice was also assessed at 4 h after FD-4 was used. The results showed that intestinal permeability was significantly decreased in infected mice treated with the inhibitors.

The inhibitors also suppressed the *T. spiralis* infection-upregulated expression of CK8, RhoA and ROCK1, restored and increased the *T. spiralis* infection-downregulated expression of TJs (E-cad, Occludin and Claudin-1). Additionally, the two inhibitors decreased the expression levels of inflammatory cytokines after *T. spiralis* infection, thereby alleviated intestinal inflammatory reaction. Histopathological examination of intestinal sections using HE and PAS staining revealed that the villi in the infected DMSO group were obviously swollen with significant structural damage, and goblet cells were increased and enlarged. Intestinal parasitic infections typically lead to thickening of the mucus layer [71]. Mucins, the main components of mucus, are secreted by goblet cells. The mucus layer covering the intestinal epithelium is usually served as a primary physical and chemical barrier. The number of goblet cells and the amount of mucin secretion are directly related to the severity of *T. spiralis* infection [37,72]. After pretreatment of mice with two inhibitors, the goblet cell number and mucin secretion significantly decreased, suggesting that both inhibitors of CK8 receptor and ROCK1 pathway effectively impeded larval invasion and clearly alleviated intestinal inflammation.

However, no inhibitors are perfectly specific. They might have off-target effects especially when they were used *in vivo*. When mice were administered by DAS (≥50 mg/kg) via gavage, the inhibitor disrupted intestinal barrier through a ROCK-independent pathway. In this study, when 20 mg/kg DAS or/and 5 mg/kg Y27632 were used, no significant impairment of intestinal barrier was observed. Previous studies showed that intraperitoneal administration of Y27632 (≥30 mg/kg) in mice caused the off-target inhibition. At 10 mg/kg, the inhibitor selectively inhibited the ROCK-MLC axis without off-target effects [73]. Therefore, 5 mg/kg Y27632 was used in this study, which obtained the expected inhibitory effect. Additionally, other studies showed that YQFM [YiQiFuMai powder injection, included three kinds of medicinal plants (*Panax ginseng*, *Ophiopogon japonicas,* and *Schisandra chinensis*) significantly increased the expression of ZO-1, Occludin and E-cad, and could alleviate the side effects caused by DAS linked with the ROCK/MLC signaling pathway [74]. Furthermore, Ruscogenin might ameliorate DAS-induced enteral barrier dysfunction via ErbB4/YAP and ROCK/MLC pathways [75]. Therefore, when a higher dose of DAS is administered in future experiment, YQFM and Ruscogenin could be used to avoid the DAS’s side effect.

In this study, the CK8 knockdown (or CK8 inhibitor DAS) and ROCK1 pathway inhibitor Y27632 treatment were used to reverse-validate the TsSPc’s action mechanism: binding of rTsSPc with CK8 receptor in gut epithelium activated the downstream RhoA/ROCK1 signaling pathway, reduced the TJs expression levels and destroyed gut epithelial barrier integrity, thereby promoted larval invasion. But the TsSPc-mediated larval invasion is also involved in other different signaling pathways that work alongside. Our previous study demonstrated that rTsSPc was also bound with RACK1 receptor and activated the MAPK/ERK1/2 signaling pathway, decreased the TJs protein expression, and facilitated larval invasion [17]. However, other studies have shown that keratin 8/18 filaments act as a “kinase sponge” that sequesters inactive MAPK modules (MKK3/6, JNK and ERK1/2) away from their substrates; when this filament network collapses (CK8 knockdown), the soluble pool of these kinases increases, leading to an apparent “activation” in Western blots without true enhancement of substrate phosphorylation [76]. These results indicated that CK8 knockdown did not lead to activation of the downstream MAPK cascade. According to the large-scale kinase-profiling data [77], 10 μM Y27632 does not significantly affect protein kinases associated with regulation of cell cycle progression, including ERK2, S6K1, GSK3β and p38MAPK isoforms, confirming that the observed effects are independent of the MAPK/ERK/1/2 cascade.

However, this study has several limitations. More precise protein-protein interaction assays, such as surface plasmon resonance (SPR) and fluorescence resonance energy transfer (FRET), are required to validate the direct interaction between TsSPc and CK8. Although the CK8 inhibitor Dasatinib (DAS) was used in this study to block rTsSPc-CK8 binding, further studies in CK8-knockout or CK8-knockdown mice are needed to precisely define the specific role of CK8 during *T. spiralis* invasion of intestinal epithelium. To further elucidate the significance of the TsSPc-CK8 interaction mechanism in the TsSPc-mediated larval invasion of gut mucosa, the *in vivo* blockade of TsSPc by passive transfer of mice with anti-TsSPc antibodies needs to be further investigated. Moreover, immunization of experimental animals with rTsSPc would produce anti-rTsSPc antibodies, which could block the binding of TsSPc with CK8 *in vivo*, thereby inhibit larval invasion and development [22,71]. But the immunoprotective efficacy of rTsSPc vaccination against *T. spiralis* larval invasion is necessary to be further evaluated in future study. In our previous mass spectrometry, keratins other than CK8 (such as keratin 18) were also identified [17]. Further experiments are required to confirm the binding and interaction of TsSPc with CK18 or other non-keratin proteins and to investigate whether they play a synergistic role during larval invasion.

In conclusion, the specific binding of rTsSPc and CK8 receptor in Caco-2 cells was verified by molecular docking, IFA, GST pull-down and Co-IP test. The results of qPCR and Western blotting showed that the binding of rTsSPc to CK8 up-regulated the CK8 expression and activated RhoA/ROCK1 pathway. Knocking down CK8 in Caco-2 cells and ROCK1 inhibitor inhibited the activation of RhoA/ROCK1 pathway, abolished the rTsSPc-reduced TJs expression, rTsSPc-increased Caco-2 monolayer permeability, and inhibited the *in vitro* larval invasion of cell monolayers. The inhibitors of CK8 receptor and ROCK1 pathway significantly inhibited the activation of CK8, RhoA and ROCK1 in intestinal epithelia of infected mice. The two inhibitors also decreased intestinal inflammatory cytokine expression, impeded larval invasion of intestinal mucosa, and alleviated intestinal inflammation. These results indicated that TsSPc binding to CK8 in intestinal epithelium activated RhoA/ROCK1 pathway, reduced the TJs expression and disrupted intestinal integrity and barrier function, therefore mediated larval invasion of host intestinal mucosa. TsSPc can be considered as a potential molecular target for vaccines against *T. spiralis* intestinal invasive stage.

## Supporting information

S1 Table Primer sequences of human and murine TJs, mucin and cytokines in qPCR. (DOCX)

S2 Table Docking score results of TsSPc and CK8 proteins. (DOCX)

S3 Table Raw data of Fig 2-Fig 13 in this study. (XLSX)

S1 Fig qPCR analysis of CK8 knockdown affecting Clauding-1 mRNA expression. Caco-2 cells were incubated with rTsSPc (20 μg/ml) for 2 h after CK8 knockdown. Cellular mRNA was extracted, and the transcription levels of Claudin-1 were assessed by qPCR, the GAPDH was used as the reference gene. The results showed that rTsSPc stimulation and CK8 knockdown had no evident effect on the expression level of Claudin-1 mRNA in Caco-2 cells.
(TIF)
