# Supplementary material for: Trichinella spiralis serine protease mediates larval invasion of gut epithelium via binding to CK8 and activating RhoA/ROCK1 pathway
Source: PLoS Negl Trop Dis. 2025 Nov 13;19(11):e0013725. doi: 10.1371/journal.pntd.0013725 (PMC12629419; doi:10.1371/journal.pntd.0013725)
Supplement: S1 Table — (DOCX) [file pntd.0013725.s001.docx]

**S1 Table. Primer sequences of human and murine TJs, mucin and cytokines in qPCR**

| Genes | Sequences (5ʹ to 3ʹ) | GenBank no. |
| --- | --- | --- |
| CK 8 | F: AGGACCTGCAGGAAGGGATCT | NM_001256282.2 |
| (Human) | R: AGGTGGACACCTTGTAGGACT |  |
| RhoA | F: TCTGTCCCAACGTGCCCATCAT | NM_001664.4 |
| (Human) | R: CTGCCTTCTTCAGGTTTCACCG |  |
| ROCK-1 | F: GAAACAGTGTTCCATGCTAGACG | NM_005406.3 |
| (Human) | R: GCCGCTTATTTGATTCCTGCTCC |  |
| E-cad | F: GCCTCCTGAAAAGAGAGTGGAAG | NM_004360.5 |
| (Human) | R: TGGCAGTGTCTCTCCAAATCCG |  |
| Occludin | F: ATGGCAAAGTGAATGACAAGCGG | XM_026274194.1 |
| (Human) | R: CTGTAACGAGGCTGCCTGAAGT |  |
| Claudin-1 | F: GTCTTTGACTCCTTGCTGAATCTG | NM_021101.5 |
| (Human) | R: CACCTCATCGTCTTCCAAGCAC |  |
| GAPDH | F: GTCTCCTCTGACTTCAACAGCG | NM_002046.7 |
| (Human) | R: ACCACCCTGTTGCTGTAGCCAA |  |
| CK8 | F: TGGAAGGACTGACCGACGAGAT | NM_031170.2 |
| (Mouse) | R: GGCACGAACTTCAGCGATGATG |  |
| RhoA | F: ACTCGGAGTCCTCGCCTTGA | NM_016802.5 |
| (Mouse) | R: AAGCTCCATCACCAACAATCAC |  |
| ROCK1 | F: CACGCCTAACTGACAAGCACCA | NM_009071.2 |
| (Mouse) | R: CAGGTCAACATCTAGCATGGAAC |  |
| E-cad  (Mouse) | F: GGTCATCAGTGTGCTCACCTCT  R: GCTGTTGTGCTCAAGCCTTCAC | NM_009864.3 |
|  |  |  |
| Occludin | F: TGGCAAGCGATCATACCCAGAG | NM_001360536.1 |
| (Mouse) | R: CTGCCTGAAGTCATCCACACTC |  |
| Claudin-1 | F: GGACTGTGGATGTCCTGCGTTT | NM_016674.4 |
| (Mouse) | R: GCCAATTACCATCAAGGCTCGG |  |
| Muc2 | F: TGTGGCCTGTGTGGGAACTTT | NM_023566.4 |
| (Mouse) | R: CATAGAGGGCCTGTCCTCAGG |  |
| Muc5ac | F: CTGTGACATTATCCCATAAGCCC | NM_010844.3 |
| (Mouse) | R: AAGGGGTATAGCTGGCCTGA |  |
| TNF-α | F: CCCTCACACTCAGATCATCTTCT | NM_013693.3 |
| (Mouse) | R: GCTACGACGTGGGCTACAG |  |
| IL-1β | F: AGCTCTCCACCTCAATGGAC | NM_008361.4 |
| (Mouse) | R: ATCATTGCGTGGGATCTTGA |  |
| IL-10 | F: CCCTTTGCTATGGTGTCCTT | NM_010548.2 |
| (Mouse) | R: TGGTTTCTCTTCCCAAGACC |  |
| TGF-β | F: TGATACGCCTGAGTGGCTGTCT | NM_011577.2 |
| (Mouse) | R: CACAAGAGCAGTGAGCGCTGAA |  |
| GAPDH | F: CATCACTGCCACCCAGAAGACTG | NM_001411840.1 |
| (Mouse) | R: ATGCCAGTGAGCTTCCCGTTCAG |  |
