# Supplementary material for: Trichinella spiralis serine protease mediates larval invasion of gut epithelium via binding to CK8 and activating RhoA/ROCK1 pathway
Source: PLoS Negl Trop Dis. 2025 Nov 13;19(11):e0013725. doi: 10.1371/journal.pntd.0013725 (PMC12629419; doi:10.1371/journal.pntd.0013725)
Supplement: S2 Table — (DOCX) [file pntd.0013725.s002.docx]

**S2 Table. Observed intermolecular interactions**

**betweenTsSPc and CK8 proteins**

| Interaction types | TsSPc | Dist.(Å) | CK8 |
| --- | --- | --- | --- |
| Hydrogen  bridges | ALA-64 | 3.5 | ILE-386 |
|  | SER-94 | 3.1 | SER-36 |
|  | VAL-133 | 3.2 | SER-34 |
|  | ASN-203 | 2.9 | GLN-370 |
|  | THR-170 | 3.2 | TYR-427 |
|  | TYR-172 | 3.2 | SER-425 |
| Salt bridges | ASP-112 | 2.6 | ARG-392 |
