# Supplementary figures and images for: Trichinella spiralis serine protease mediates larval invasion of gut epithelium via binding to CK8 and activating RhoA/ROCK1 pathway
Source: PLoS Negl Trop Dis. 2025 Nov 13;19(11):e0013725. doi: 10.1371/journal.pntd.0013725 (PMC12629419; doi:10.1371/journal.pntd.0013725)

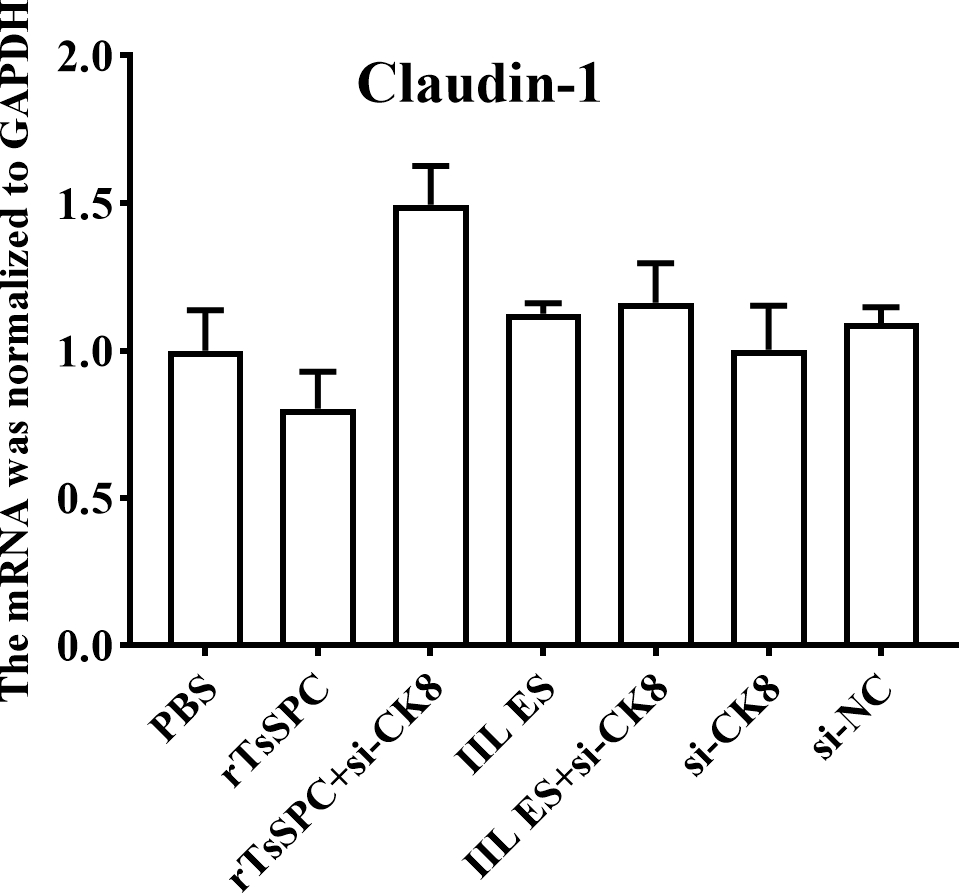

Supplement: S1 Fig — Caco-2 cells were incubated with rTsSPc (20 μg/ml) for 2 h after CK8 knockdown. Cellular mRNA was extracted, and the transcription levels of Claudin-1 were assessed by qPCR, the GAPDH was used as the reference gene. The results showed that rTsSPc stimulation and CK8 knockdown had no evident effect on the expression level of Claudin-1 mRNA in Caco-2 cells. (TIF) [file pntd.0013725.s004.tif]
